# The Type III Secretion System (T3SS) of *Escherichia Coli* Promotes Atherosclerosis in Type 2 Diabetes Mellitus

**DOI:** 10.1002/advs.202413296

**Published:** 2025-01-14

**Authors:** Yao‐Yuan Zhang, Song‐Tao Chen, Gang Chen, Le Zhou, Guo‐Liang Zhou, Xin‐Yuan Yu, Long Yuan, Wei‐Qian Deng, Zhen‐Bo Wang, Jing Li, Yi‐Fu Tu, Da‐Wei Zhang, Yuan li, Abdul Sammad, Xiao Zhu, Kai Yin

**Affiliations:** ^1^ Department of General Practice The Fifth Affiliated Hospital of Southern Medical University Guangzhou 510515 China; ^2^ Guangdong Provincial Key Laboratory of Drug Non‐Clinical Evaluation and Research Guangzhou 510515 China; ^3^ Guangxi Key Laboratory of Diabetic Systems Medicine Guilin Medical University Guilin 541199 China; ^4^ Department of Cardiology The Second Affiliated Hospital of Guilin Medical University Guilin 541199 China; ^5^ Department of Imaging Diagnosis Zhujiang Hospital of Southern Medical University Guangzhou 510515 China; ^6^ Group on the Molecular and Cell Biology of Lipids and Department of Pediatrics Faculty of Medicine and Dentistry University of Alberta Edmonton Alberta T6G 2R3 Canada; ^7^ Guangzhou Key Laboratory of Metabolic remodeling and Precise Prevention and Control of Diabetes Guangzhou 510515 China; ^8^ Guangxi Clinical Research Center for Diabetes and Metabolic Diseases The Second Affiliated Hospital of Guilin Medical University Guilin 541199 China

**Keywords:** atherosclerosis, ferroptosis, intestinal barrier, phosphatidylcholine, T2DM, T3SS

## Abstract

Large‐scale studies indicate a strong relationship between the gut microbiome, type 2 diabetes mellitus (T2DM), and atherosclerotic cardiovascular disease (ASCVD). Here, a higher abundance of the type III secretion system (T3SS) virulence factors of *Enterobacteriaceae*/*Escherichia*‐*Shigella* in patients with T2DM‐related‐ASCVD, which correlates with their atherosclerotic stenosis is reported. Overexpression of T3SS via *Citrobacter rodentium* (CR) infection in Apoe‐/‐ T2DM mice exacerbated atherosclerotic lesion formation and increased gut permeability. Non‐targeted metabolomic and proteomic analysis of mouse serum showed that T3SS caused abnormal glycerophospholipid metabolism in mice. Proteomics, RNA sequencing, and functional analyses showed that T3SS induced ferroptosis in intestinal epithelial cells, partly due to increased expression of ferritin heavy chains (FTH1). This findings first demonstrated that T3SS increases ferroptosis in intestinal epithelial cells, via disrupting the intestinal barrier and upregulation of phosphatidylcholine, thereby exacerbating T2DM‐related ASCVD.

## Graphical abstract

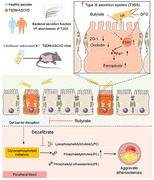

## 1 Introduction

The global prevalence of type 2 diabetes mellitus (T2DM) among people aged 20–79 years was 10.5% (536.6 million people) in 2021, rising to 12.2% (783.2 million) in 2045. T2DM is associated with premature atherosclerotic cardiovascular disease (ASCVD).^[^ ^1^ ^]^ Patients with T2DM typically develop severe cardiovascular abnormalities 14.6 years earlier than individuals without T2DM.^[^ ^2^ ^]^ Globally, ASCVD has become a leading cause of morbidity and mortality in patients with T2DM. The impact of diabetes on ASCVD results primarily from chronic hyperglycemia, increased oxidative stress, and elevated levels of advanced glycation end products, thereby creating a persistent inflammatory microenvironment that promotes ASCVD development.^[^ ^3^ ^]^ Recent trials involving thousands of patients with T2DM have identified various glucose‐lowering therapies that improve cardiovascular outcomes in a small subset of patients,^[^ ^4^ ^]^ suggesting the pathogenesis of T2DM‐accelerated ASCVD remains to be exclusive.

Substantial evidence suggests that gut flora dysbiosis is closely correlated with the occurrence and progression of T2DM and its related complications. Individuals with obesity and T2DM often have lower microbial diversity and an increased abundance of potentially pathogenic Gram‐negative bacteria, including *Proteobacteria*.^[^ ^5^ ^]^ Emerging evidence indicates that the gut microbiome in patients with ASCVD deviates from a healthy state due to an increased abundance of pathogenic bacteria, such as *Enterobacteriaceae* and *Streptococcus* spp.^[^ ^6^ ^]^ The proliferation of pathogenic bacteria increases the concentration of substances such as trimethylamine N‐oxide and lipopolysaccharide (LPS), aggravating T2DM, ASCVD, and intestinal barrier damage.^[^ ^7^ ^]^ Moreover, translocation of viable bacteria or bacterial structural components (e.g., endotoxins) into the bloodstream can lead to low‐grade systemic inflammation, thereby exacerbating atherosclerosis. Although many studies have investigated the role of intestinal microbiota in the development of T2DM or ASCVD, few studies have explored the exact mechanisms underlying the progression of T2DM‐related ASCVD. The type III secretion system (T3SS) is a key virulence component of gut pathogenic bacteria.^[^ ^8^ ^]^ Recent evidence indicates that pathogenic bacteria in the intestinal lumen, e.g., enteropathogenic *Escherichia coli* (EPEC) and enterohemorrhagic *Escherichia coli* (EHEC), utilize T3SS to act on host cells, causing intestinal damage.^[^ ^9^ ^]^ Children with Prader–Willi syndrome, who have higher expression of virulence factors of the gut flora, such as T3SS, show more serious host inflammation.^[^ ^10^ ^]^ Metagenomic analysis of the intestinal microbiota of patients with ankylosing spondylitis revealed increased T3SS abundance in the proinflammatory intestinal microbiota.^[^ ^11^ ^]^ However, it remains unknown whether intestinal T3SS contribute to the progress of T2DM‐related ASCVD.

Here, to better understand the origins of atherosclerosis aggravated by T2DM, we focus on changes in the gut microbiome induced by type 2 diabetes and determined that increased T3SS abundance in type 2 diabetes is associated with increased atherosclerotic lesions. We provide evidence for how gut microbiota T3SS functionally contributes to type 2 diabetes‐associated atherosclerosis. We tested the hypothesis that increased T3SS abundance under type 2 diabetic conditions may lead to intestinal barrier dysfunction and abnormal glycerophospholipid metabolism, potentially resulting in atherosclerosis.

## 2 Results

### 2.1 The Abundance of T3SS of Pathogenic *Escherichia Coli (E. Coli)* Is Associated With Atherosclerosis Progression in Patients With T2DM

**Figure 1:**
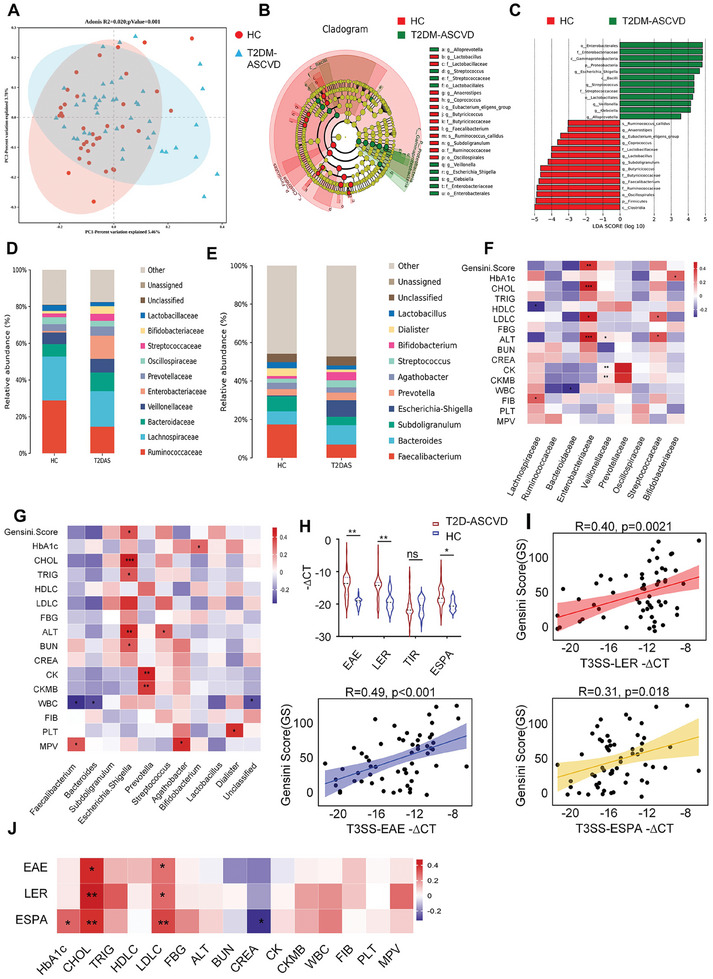
The Abundance of T3SS of Pathogenic *Escherichia Coli* (*E. Coli*) Is Associated With Atherosclerosis Progression in Patients With T2DM. A) Overall gut microbial structure in healthy controls (HC) (*n* = 35) and patients with T2DM‐ASCVD (*n* = 45). B) Partial Least Squares Discriminant Analysis (PLS‐DA) calculated using Unweighted_unifrac at the OTU‐level significantly separated non‐responders from HC and T2D‐AS. C) Using linear discriminant analysis (LEfSe, LDA>3.0), differential OTUs responsible for the discrimination between two groups were identified. These enriched OTUs in T2DAS group primarily belonged to Enterobacterales, Enterobacteriaceae, Gammaproteobacteria and Escherichia_Shigella; and depleted OTUs were mainly assigned to Clostridia and Firmicutes. D,E) Family and genus levels of microbial composition in HC and patients with T2DM‐ASCVD. F) Spearman's correlation between diabetic atherosclerosis‐related family and patients' atherosclerosis severity and serum indices. Correlation coefficients are reported as Spearman correlations. G) Spearman's correlation between diabetic atherosclerosis‐related genera and patients' atherosclerosis severity and serum indices. Correlation coefficients are reported as Spearman correlations. H) qRT‐PCR analysis of the abundance of T3SS in fecal samples(healthy controls (HC) (*n* = 23) and patients with T2DM‐ASCVD (*n* = 58)). I) Scatterplots indicated correlation of Gensini Score with fecal T3SS abundance (EAE, LER, ESPA). Correlation coefficients are reported as Spearman correlations. J) Scatterplots indicated correlation of TRIG, CHOL, and LDLC alt with fecal LER abundance. Correlation coefficients are reported as Spearman correlations. All data are expressed as mean ± SEM, analyzed by an unpaired two‐sided t‐test. *P < 0.05, **P < 0.01, ***P < 0.001, ****P < 0.0001.

To investigate the relationship between gut microbiota alterations and atherosclerosis progression in patients with T2DM, we recruited 45 patients and 35 healthy individuals. The baseline demographic and clinical characteristics of these patients are shown in **Table** **1**. Bacterial DNA was extracted for the overall profile of all fecal microbiomes in both cohorts using 16S rRNA gene sequencing (Figure S1A, Supporting Information). Principal component analysis (PCA) was used to determine the microbiome composition of different samples. The β‐diversity differed significantly between both groups (**Figure** **1**A). To further explore potential confounding factors within our study, additional PCA analyses were performed to evaluate the influence of the use of medications such as antihypertensive, lipid‐lowering, and antidiabetic agents on the gut flora composition. The results, illustrated in Figure S1 (Supporting Information), indicate that these medications do not significantly differentiate participant groups, indicating a minimal influence of these factors on our study outcomes (Figure S1B, Supporting Information). Linear discriminant analysis of effect size and random forests for assessing bacterial communities revealed a marked difference between the two study groups (Figure 1B,C; Figure S1C–E, Supporting Information). Levels of members at certain families (e.g., *Enterobacteriaceae*, *Streptococcaceae*) and genera (e.g., *Escherichia, Shigella*, *Streptococcus*, *Veillonella*, *Klebsiella*, *Alloprevotella*) were more abundant in patients with T2DM‐ASCVD than in healthy participants (Figure 1C). To further identify differences in microbial communities, we used operational taxonomic unit abundance to segregate groups of microbiomes. At the family level, *Enterobacteriaceae* showed the greatest increase in patients with T2DM‐ASCVD. Similarly, at the genus level, *Escherichia‐Shigella*, a member of the *Enterobacteriaceae* family, was most elevated in these patients compared with that in the control group (Figure 1D,E). Notably, in patients with T2DM‐ASCVD, *Enterobacteriaceae* had the highest positive correlation with the Gensini score,^[^ ^12^ ^]^ describing atherosclerotic stenosis, and with serum low‐density lipoprotein (LDL‐C) and cholesterol levels (Figure 1F). *Escherichia*‐*Shigella* also showed the strongest positive correlation with the Gensini score, serum cholesterol, and serum triglyceride levels in these patients (Figure 1G). Therefore, the increase in *Enterobacteriaceae*/*Escherichia*‐*Shigella* in the intestinal flora may be associated with the development of atherosclerosis in patients with T2DM‐ASCVD.

**Table 1:** Characteristics of the study cohort.

Variables	Patients With T2DM‐ASCVD [n = 45]	Controls Without T2DM‐ASCVD [n = 35]
Gender	24(53.3%)	17(48.5%)
Age (year)	64.8 ± 9.89	65.48 ± 9.79
Height (cm)	160.7 ± 6.91	161.5 ± 8.37
Weight (kg)	63.6 ± 12.09	59.7 ± 12.42
Body Mass Index (BMI)	24.8 ± 3.49	22.7 ± 3.29
Smoking	14(31.11%)	15(25.86%)
Diastolic Blood Pressure (mmHg)	79.44 ± 10.84	74.08 ± 8.64
Systolic Blood Pressure (mmHg)	135.33 ± 22.00	119.34 ± 9.91
Hypertension	33(73.33%)	2(5.71%)
T2DM	45(100%)	/
Gensini Score (GS)	57.44±38.06	/
Number of diseased Coronary Artery	3.37 ± 0.71	/
Metformin	21(42%)	/
Acarbose	35(70%)	/
Insulin	12(24%)	/
Aspirin	32(64%)	/
Atorvastatin	33(66%)	/
PPI	/	/
Captopril	10(20%)	/
Irbesartan	16(32%)	/
Nifedipine	13(26%)	/
WBC(10^9^/L)	8.79 ± 2.88	6.53 ± 1.89
TRIG (mmol/L)	2.32 ± 2.15	1.50 ± 1.06
PLT(10^9^/L)	243.82 ± 98.1	274.11 ± 55.03
MPV(FL)	10.44 ± 0.9	10.20 ± 0.74
LDLC (mmol/L)	2.69 ± 1.32	3.02 ± 0.72
HDLC (mmol/L)	1.03 ± 0.26	1.50 ± 0.37
HbA1c (%)	7.75 ± 1.64	5.21 ± 0.44
FIB(g/L)	3.75 ± 1.11	/
FBG	8.09 ± 2.37	5.05 ± 0.40
CREA (umol/L)	93.55±34.141	63.42 ± 12.77
CKMB (U/L)	64.04 ± 195.22	/
CK (U/L)	423.52 ± 1335.74	/
CHOL (mmol/L)	4.43 ± 1.73	4.71 ± 0.84
BUN (nmol/L)	6.93 ± 3.35	4.40 ± 0.71
ALT (U/L)	24.5 ± 28.6	23.98 ± 24.48

Data are presented as mean ± SD or as number (%) affected.

*Escherichia*‐*Shigella* damages the intestinal barrier by adhering to epithelial cells. Interestingly, our level two or three Kyoto Encyclopedia of Genes and Genomes (KEGG) analyses showed that compared with that in healthy participants, metabolic pathways with high abundance in the microbiome of patients with T2DM‐ASCVD were mainly enriched in cell growth and death, digestive system, and infectious disease (Figure S1F, Supporting Information). Moreover, bacterial invasion of epithelial cells, shigellosis, and pathogenic *Escherichia Coli (E. Coli)* infection pathways were enriched in the T2DM‐ASCVD group (Table S1, Supporting Information). Given that pathogenic *E. coli* are members of *Escherichia*‐*Shigella* at the species level, we hypothesized that pathogenic *E. coli* plays an important role in T2DM‐ASCVD progression. Therefore, we selected 58 patients with T2DM‐ASCVD and 23 healthy individuals and matched the two groups in terms of age, sex, and body mass index (**Table** **2**). Quantitative polymerase chain reaction (qRT‐PCR) analysis using bacterial DNA to detect the main species of pathogenic *E. coli*, including EHEC and EPEC,^[^ ^13^ ^]^ confirmed a significantly increased abundance of EHEC and EPEC in patients with T2DM‐ASCVD (Figure S1E, Supporting Information). T3SS are key virulence factors of Gram‐negative pathogens, including pathogenic *E. coli*, which cause intestinal diseases, such as diarrhea^[^ ^14^ ^]^ and inflammatory bowel disease in humans.^[^ ^15^ ^]^ We used qRT‐PCR to measure the expression of the crucial T3SS components (*eae*, *ler*, *tir*, and *espA*) in the feces of patients with T2DM‐ASCVD,^[^ ^16^ ^]^ and found that their levels were significantly elevated in patients with T2DM‐ASCVD compared with those in healthy individuals (Figure 1H), and their abundance in the intestinal flora was positively correlated with the Gensini score and plasma cholesterol and LDL‐C levels (Figure 1I,J), suggesting the abundance of T3SS of Pathogenic *E. Coli* may serve as predictors of T2DM‐ASCVD progression.

**Table 2:** Characteristics of the study cohort.

Variables	Patients With T2DM‐ASCVD [n = 58]	Controls Without T2DM‐ASCVD [n = 23]
Gender	27(46.55%)	17(52.17%)
Age (year)	62.56 ± 9.16	59.17 ± 9.79
Height (cm)	161.44 ± 7.99	163.47 ± 8.37
Weight (kg)	66.63 ± 10.71	65.03 ± 10.06
Body Mass Index (BMI)	25.50 ± 3.27	24.24 ± 2.68
Smoking	21(36.2%)	6(26.08%)
Diastolic Blood Pressure (mmHg)	82.65 ± 13.31	76.78 ± 10.75
Systolic Blood Pressure (mmHg)	141.98 ± 24.21	126 ± 14.53
Hypertension	35(60.34%.)	2(8.69%)
T2DM	58(100%)	/
Gensini Score (GS)	54.24 ± 32.88	/
Number of diseased Coronary Artery	2.81 ± 0.78	/
Metformin	31(53%)	/
Acarbose	42(72%)	/
Insulin	17(29%)	/
Aspirin	39(67%)	/
Atorvastatin	77(75%)	/
PPI	/	/
Captopril	18(31%)	/
Irbesartan	16(27%)	/
Nifedipine	17(29%)	/
WBC(10^9/L)	7.85 ± 2.36	6.02 ± 1.39
TRIG (mmol/L)	2.55 ± 2.32	1.32 ± 0.72
PLT(10^9/L)	256.4 ± 102.45	267.73 ± 64.08
MPV(FL)	10.23 ± 1.56	10.39 ± 0.71
LDLC (mmol/L)	2.76 ± 1.15	3.05 ± 0.79
HDLC (mmol/L)	1.04 ± 0.20	1.38 ± 0.31
HbA1c (%)	7.39 ± 1.19	5.21 ± 0.44
FIB(g/L)	3.52 ± 1.24	/
FBG	8.05 ± 2.34	5.37 ± 0.36
CREA (umol/L)	89.56 ± 30.62	72.56 ± 16.64
CKMB (U/L)	41.48 ± 77.09	/
CK (U/L)	321.43 ± 605.08	/
CHOL (mmol/L)	4.51 ± 1.37	4.73 ± 0.92
BUN (nmol/L)	7.94 ± 7.41	4.74 ± 0.97
ALT (U/L)	23.01 ± 13.03	20.61 ± 16.92

Data are presented as mean ± SD or as number (%) affected.

### 2.2 The T3SS of Pathogenic *E. Coli* Mediates the Aggravation of Atherosclerosis in T2DM

**Figure 2:**
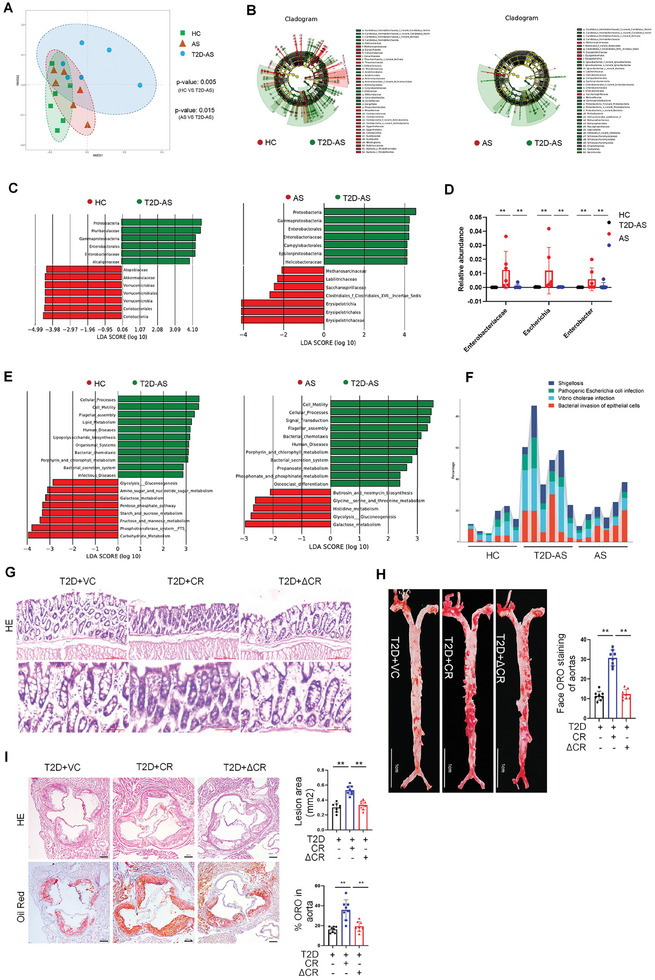
The T3SS of Pathogenic E. Coli Mediates the Aggravation of Atherosclerosis in T2DM. A) Principal coordinates analysis (PCoA) plots of the fecal microbiota based on Bray‐Curtis distances in different groups, each group is represented by different color(*n* = 6 mice per group). B) Cladogram. The circles represent the phylum, class, order, family, and genus from the inside to the outside. Each small circle at different classification levels represents a classification at that level. The diameter of the small circle is proportional to the relative abundance. C) LDA score map. The histogram's length represents the LDA value's size(LDA>2, *p* < 0.05). D) Relative abundance of Enterobacteriaceae, Escherichia and Enterobacter(*n* = 6 mice per group). E) LDA score map. KEGG pathways. F) KEGG Level 3 gene relative abundance histogram. G) Hematoxylin and eosin staining of the colon sections (*n* = 8 mice per group). Scale bars, 300 µm or 100 µm. H) Oil Red O staining of enface atherosclerotic lesions in aortas from indicated groups (*n* = 8 mice per group). Scale bars, 1 cm. I) Oil Red O staining on aortic root sections and representative images of hematoxylin and eosin (HE) from indicated mice. (*n* = 8 mice per group). Scale bars, 200 µm. All data are expressed as mean ± SEM, analyzed by an unpaired two‐sided t‐test. *P < 0.05, **P < 0.01, ***P < 0.001, ****P < 0.0001.

In order to investigate the exact mechanism of microbiota changes during the development of atherosclerosis (AS) associated with type 2 diabetes mellitus (T2D), we verified that microbiota changes during the development of T2DM affect the occurrence and development of atherosclerosis in mice. ApoE−/− mice were fed a normal chow diet (HC), a high fat diet (AS) or a high fat diet with intraperitoneal injection of streptozotocin (STZ) (T2D‐AS).^[^ ^17^ ^]^ The metagenomic sequencing of stool samples were performed to investigate the alteration of gut microbiota from HC, AS and T2D‐AS mice (8 week). Fecal microbiota principal coordinate analysis (PCoA) was employed to assess the overall structural differences among HC, AS and T2D‐AS groups (**Figure** **2**A). As shown in Figure 2B,C, distinct clustering patterns based on group affiliation were observed among the three groups. Additionally, linear discriminant analysis (LDA) effect size (LEfSe) was utilized to identify species with significant differences in abundance between groups. The abundance of *Akkermansiaceae, Verrucomicrobiae*, and *Coriobacteriales* was notably increased in the HC group, while the abundance of *Labilitrichaceae, Saccharospirillaceae*, and *Erysipelotrichaceae* was significantly increased in the AS group, whereas *Proteobacteria, Gammaproteobacteria, Enterobacterales*, and *Enterobacteriaceae* were the most significantly increased abundant species in the T2D‐AS group (Figure 2B,C; Figure S2A,B, Supporting Information). Analysis of detailed changes in microbiota composition at the genus level revealed a significantly increased abundance of *Enterobacteriaceae, Escherichia*, and *Enterobacter* in the T2D‐AS group compared with that in the HC and AS groups (Figure 2D). Further analysis at the species level found that compared with that in the HC group and AS group, the relative abundance of *E. Coli* and *Akkermanophilus* in the T2D‐AS group was significantly increased and decreased, respectively (Figure S2C, Supporting Information). KEGG pathway analysis showed that the gut bacterial function in T2D‐AS mice differed from that in HC and AS mice with a significant increase in the flagellar assembly, lipopolysaccharide biosynthesis, and bacterial secretion system (Figure 2E). Bacterial invasion of epithelial cells, *Vibrio cholerae* infection, pathogenic *E. Coli* infection, and Shigellosis was also significantly enriched in the T2D‐AS group (Figure 2F). As expected, the virulence factors (VF) abundance, which was most related to T3SS (Table S2, Supporting Information) in pathogenic bacteria above mentioned, was higher in the T2D‐AS group than in the HC and AS groups. Taking these data together, we found that diabetes‐induced intestinal microbiome dysregulation occurred before diabetes‐related atherosclerosis.

To further verify the exact role of the T3SS in T2D related‐AS, we established a T2D‐AS mice model using germ‐free ApoE‐/‐ mice.^[^ ^18^ ^]^ EHEC does not cause enteric disease in rodents. The natural mouse pathogen *Citrobacter rodentium*, which employs a LEE‐encoded T3SS similar to EHEC for intestinal colonization, has been used extensively as a model for EHEC infection.^[^ ^19^ ^]^ Thus, we employed the *Citrobacter rodentium* mouse infection model to investigate increases bacterial T3SS virulence in the occurrence and development of atherosclerosis. Mice received vehicle control, wild‐type *Citrobacter rodentium* (WT CR), the murine equivalent to human EPEC/EHEC, or ΔescN CR (a *Citrobacter rodentium* mutant with defective T3SS function)^[^ ^8^ ^]^ by oral gavage. The abundance of T3SS components in the feces of T2D mice administered with WT CR was significantly increased compared with that in normal mice receiving WT CR (Figure S2D, Supporting Information). First, we found that there was no significant difference in intestinal colonization between WT CR and ΔescN CR in mice. In addition, infection with WT CR led to chronic intestinal inflammation rather than acute intestinal inflammation in mice, whereas infection with ΔescN CR did not lead to chronic intestinal inflammation in mice (Figure 2G; Figure S2E, Supporting Information). Notably, infection with WT CR but not ΔescN CR resulted in a significant increase in atherosclerotic plaque area compared with that in vehicle control (Figure 2H,I; Figure S2F, Supporting Information). These results suggest that elevated T3SS level in the intestine accelerates atherosclerosis in T2D‐AS mice.

### 2.3 Pathogenic *E. Coli* T3SS Causes Intestinal Barrier Damage to Accelerate Atherosclerosis in T2DM

**Figure 3:**
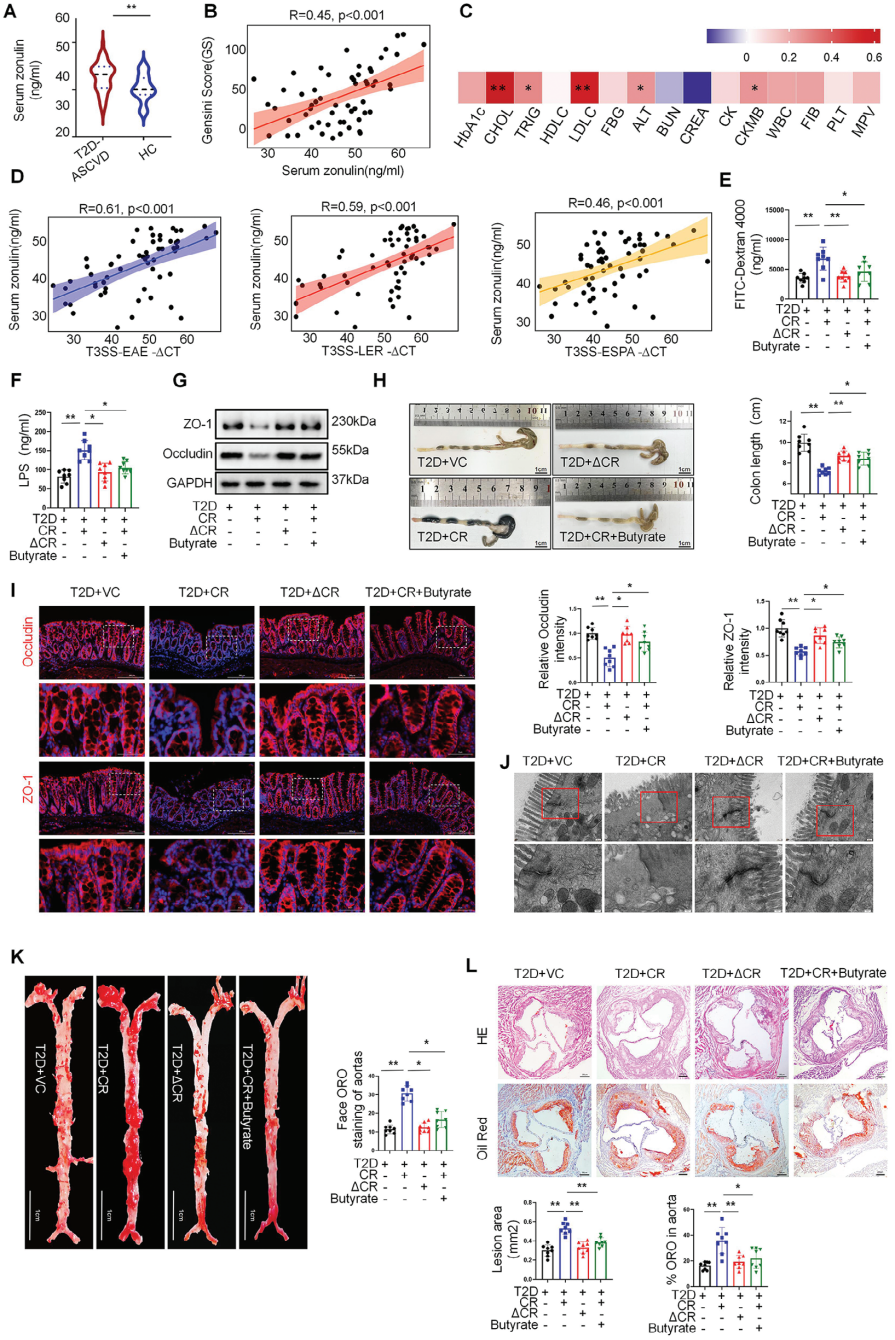
A) Pathogenic *E. Coli* T3SS Causes Intestinal Barrier Damage to Accelerate Atherosclerosis in T2DM. Serum zonulin in the T2DM‐ASCVD group (*n* = 58) and HC group (*n* = 23). B) Scatterplots indicating correlation of serum zonulin with Gensini Score (*n* = 58). Correlation coefficients are reported as Spearman correlations. C) Scatterplots indicating correlation of fecal EAE abundance with blood test index (patients with T2DM‐ASCVD (*n* = 58)). Correlation coefficients are reported as Spearman correlations. D) Scatterplots indicating correlation of serum zonulin with fecal T3SS abundance (EAE, LER, ESPA) (*n* = 58). Correlation coefficients are reported as Spearman correlations. E) Intestinal permeability assay, Plasma DX‐4000–FITC (mg/ml) oral‐challenge measured mice control, wild‐type CR, ΔescN CR and butyrate treatment CR (*n* = 8 mice per group). F) Serum LPS content of mice (*n* = 8 mice per group). G) Western blot result and quantification of the Occludin and ZO‐1 protein levels in the colon (*n* = 8 mice per group). H) Statistics of colon length (*n* = 8 mice per group). Scale bars, 1 cm. I) Representative immunofluorescence staining for ZO‐1 and occludin in mice fed with control, wild‐type CR, ΔescN CR, and butyrate treatment CR. Immunofluorescence quantization of proteins in each group (*n* = 8 mice per group). Scale bars, 200 µm or 50 µm. J) Representative transmission electron microscope images of mitochondria in the group (*n* = 8 per group). Scale bars, 200 nm or 100 nm. K) Oil Red O staining of enface atherosclerotic lesions in aortas from indicated groups (*n* = 8 mice per group). Scale bars, 1 cm. L) Oil Red O staining on aortic root sections and representative images of hematoxylin and eosin (HE) from indicated mice.(*n* = 8 mice per group). Scale bars, 200 µm. All data are expressed as mean ± SEM, analyzed by an unpaired two‐sided t‐test. *P < 0.05, **P < 0.01, ***P < 0.001, ****P < 0.0001.

Given the pathogenic function of T3SS in intestinal barrier damage and the important role of intestinal barrier damage in aggravating atherosclerosis progression, we hypothesized that intestinal barrier impairment is a potential link between T3SS and T2DM‐ASCVD progression. We indeed observed that the expression of zonulin, a marker protein of intestinal barrier damage,^[^ ^20^ ^]^ was significantly higher in patients with T2DM‐ASCVD than in healthy controls (**Figure** **3**A), and was positively correlated with the Gensini score (Figure 3B) and plasma LDL‐C and cholesterol levels in patients with T2DM‐ASCVD (Figure 3C). Moreover, the abundance of *eae*, *ler*, *tir*, and *espA*, the structural and effector genes of T3SS, were positively correlated with zonulin levels (Figure 3D).

Next, to verify whether restoring intestinal barrier function can abolish the progression of atherosclerosis in T3SS‐induced ApoE‐/‐ T2D mice, we intervened with butyrate, an intestinal barrier protector. Next, we examine whether the intestinal barrier function and the progression of atherosclerosis of ApoE‐/‐ T2D mice with WT CR infection can be restored by butyrate administration. Mice infected with WT CR had reduced colon length, while mice infected with ΔescN CR displayed no difference in colon length compared with mice treated with vector control. Butyrate administration alleviated the shortening of colon length in CR‐infected mice (Figure 3H; Figure S3A, Supporting Information). We then used FITC‐dextran to evaluate the intestinal permeability of the proximal and distal colon of T2D‐AS mice receiving different treatments. The colon of mice infected with WT CR exhibited a higher intestinal permeability than that of vector‐treated control mice, and the increased paracellular permeability was substantially attenuated by butyrate supplementation. On the other hand, the colonic permeability of mice infected with ΔescN CR was comparable to that of vector‐treated control mice and was lower than that of WT CR‐infected mice (Figure 3E,F). Intestinal permeability is regulated by multiple factors, including physical barrier function. We then detected the expression of physical barrier proteins, occludin and zona occludens‐1 (ZO‐1),^[^ ^21^ ^]^ in the colon. Immunohistochemistry, western blots, and immunofluorescence data showed that infection with WT CR but not ΔescN CR significantly reduced the expression of occludin and ZO‐1, which was restored by butyrate supplementation (Figure 3I; Figure S3C, Supporting Information). Similar changes were observed in the mRNA levels of occludin and ZO‐1 (Figure S3B, Supporting Information). Analysis of the ultrastructure of mouse colonic compact junctions (TJs) using transmission electron microscopy (TEM) revealed that control and ΔescN CR‐infected mice had comparable intact barrier structures. In contrast, the colon of mice infected with WT CR displayed dilation of intercellular space, indicating an impaired barrier. This damage, however, was substantially rescued by butyrate supplementation (Figure 3J). Overall, these results indicate that the presence of T3SS in pathogenic *E. coli* increases colonic permeability and impairs the intestinal barrier in T2D‐AS mice.

Gross examination revealed that butyrate treatment significantly reduced atherosclerotic lesions in the aortic arch of mice receiving WT CR (Figure 3K,L). Lipid deposition and plaque area in atherosclerotic lesions of WT CR‐treated mice were also significantly reduced by butyrate treatment, as evidenced by Oil red O and hematoxylin‐eosin staining of aortic roots (Figure 3K,L). Collectively, our data suggest that aberrantly upregulated T3SS virulence factors promote T2D‐AS progression, at least in part by causing intestinal barrier damage.

### 2.4 T3SS Damages the Intestinal Barrier, Leads to Abnormal Phosphatidylcholine and Lipid Metabolism, and Accelerates Atherosclerosis in T2D

**Figure 4:**
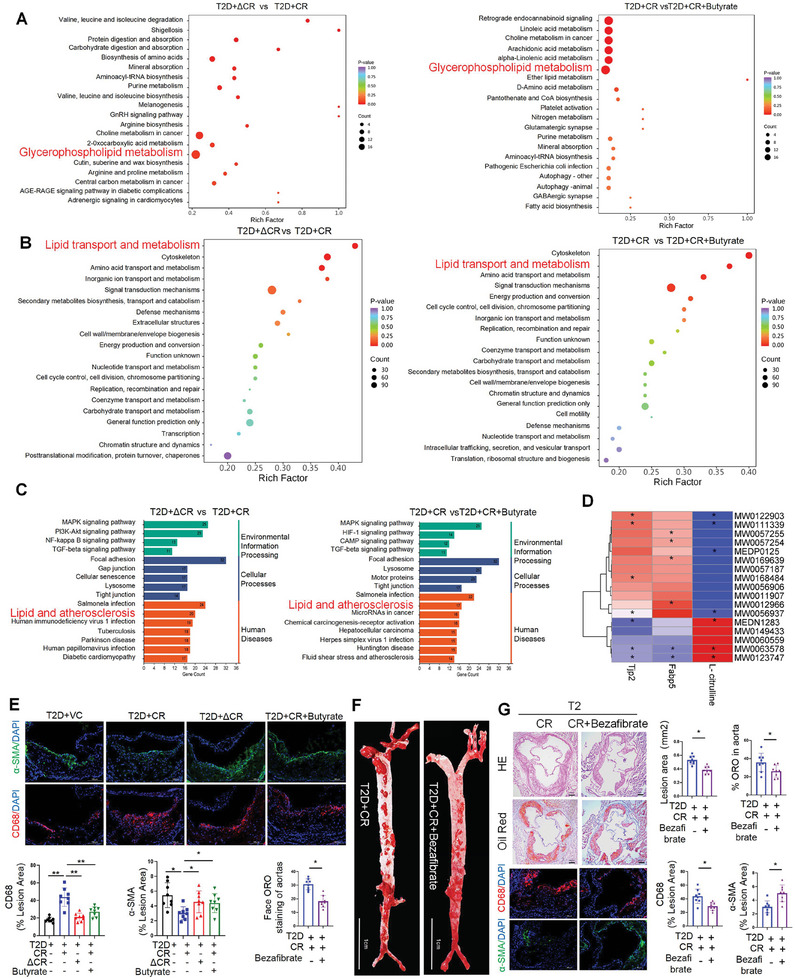
T3SS Damages The Intestinal Barrier Leads To Abnormal Phosphatidylcholine And Lipid Metabolism Accelerate Atherosclerosis in T2DM. A) Pathway enrichment analysis performed using the significantly different serum metabolites (*n* = 4 mice per group). B) Pathway enrichment analysis performed using the significantly different serum proteins (*n* = 4 mice per group). C) GO analysis of the proteins in serum (*n* = 4 mice per group). D) Correlation analysis of serum intestinal barrier related proteins and metabolites related to serum glycerophospholipid metabolism (*n* = 4 mice per group). E) Immunostaining for smooth muscle actin (SMA) and F4/80 on aortic root slides collected from indicated mice. Quantification of atherosclerosis lesion areas in each group (*n* = 8 mice per group). Scale bars, 100 µm. F) Oil Red O staining of enface atherosclerotic lesions in aortas from indicated groups (*n* = 8 mice per group). Scale bars, 1 cm. G) Oil Red O staining on aortic root sections and representative images of hematoxylin and eosin (HE) from indicated mice, Scale bars, 200 µm. Immunostaining for smooth muscle actin (SMA) and F4/80 on aortic root slides collected from indicated mice. Quantification of atherosclerosis lesion areas in each group. (*n* = 8 mice per group). Scale bars, 100 µm. All data are expressed as mean ± SEM, analyzed by an unpaired two‐sided t‐test. *P < 0.05, **P < 0.01, ***P < 0.001, ****P < 0.0001.

To comprehensively understand the mechanism of T3SS‐induced intestinal barrier damage and host metabolic changes, we performed serum metabolomics and proteomic analyses. Untargeted metabolomics was conducted on mouse serum samples from three groups. The results of the PCA score map of serum metabolites showed that the metabolic composition of the WT CR group was significantly different from that of the ΔescN CR or the WT CR plus butyrate treatment groups, indicating that T3SS‐induced intestinal barrier damage caused a significant change in the host metabolite composition (Figure S4A,B, Supporting Information). The metabolites identified were mainly glycerol phospholipid, glycerides, alkaloids, glycerides, and aldehyde. Furthermore, in the comparison of ΔescN CR and WT CR, pathway enrichment analysis showed that the major altered pathway included Shigellosis and glycerophospholipid metabolic pathway, while the glycerophospholipid metabolic pathway was enriched when comparing WT CR with WT CR plus butyrate groups. Heat map results showed that serum glycerophospholipid metabolites, including phosphatidyl ethanolamine, phosphatidylcholine, lysophosphatidic ethanolamine, and phosphatidic acid, were significantly increased in WT CR‐treated mice compared with those in ΔescN CR or WT CR plus butyrate‐treated mice (**Figure** **4**A; Figure S4C, Supporting Information).

Considering that circulating proteins can affect pathophysiological changes in mice, we conducted proteomics analysis on serum samples from ΔescN CR, WT CR, and WT CR plus butyrate mice (Figure S4A,B, Supporting Information). KEGG analysis showed that pathways in ΔescN CR and WT CR mice were mainly enriched for lipid transport and metabolism, cytoskeleton, and amino acid transport and metabolism. In contrast, the main enrichment pathways in WT CR plus butyrate and WT CR mice included cytoskeleton, lipid transport and metabolism, and signal transduction mechanisms, among which lipid transport and metabolism was the most significantly enriched (Figure 4B,C). Heat map analysis of proteins related to lipid transport and metabolic pathways showed that Plcg2, Lysophospholipase 2, and fatty acid‐binding protein 5 (FABP5) were significantly increased in the WT CR group compared with those in the ΔescN CR group. In addition, we found that proteins associated with tight junctions were significantly altered in the WT CR group compared with those in the ΔescN CR and WT CR plus butyrate groups (Figure S4C,D, Supporting Information).

Phosphatidylcholine, trimethylamine, and choline can promote obesity, atherosclerosis, and cardiovascular disease.^[^ ^22^ ^]^ To explore the correlation between T3SS‐induced intestinal barrier damage and metabolism, we performed Spearman correlation analysis and found that serum markers of intestinal barrier damage were strongly correlated with the serum content of phosphatidylcholine, trimethylamine, and choline (Figure 4D; Figure S4G,K, Supporting Information). In addition, the heat map results of inflammation‐related proteins showed that serum pro‐inflammatory factors, such as MAPK and major urinary proteins, were significantly increased in the WT CR group compared with those in the ΔescN CR and WT CR plus butyrate groups (Figure 4C). Serum trimethylamine and choline can induce macrophage activation and promote atherosclerosis.^[^ ^23^ ^]^ Thus, we further characterized plaques by analyzing inflammatory markers and lesion stability. We found that T3SS significantly reduced intraplaque smooth muscle cell content and increased macrophage infiltration, as evidenced by reduced α‐SMA staining and increased CD68+ staining. This phenotype in WT CR mice was significantly reversed by butyrate treatment (Figure 4E). Together, these data indicate that T3SS can dilate and compromise the intestinal barrier. Aggravated glycerol phospholipid metabolism and lipid transport and metabolism in WT CR mice result in increased serum phosphatidylcholine, trimethylamine, and choline content. This may lead to low systemic inflammation (Figure S4L, Supporting Information). To further determine whether abnormal increases in phosphatidylcholine contribute to atherosclerosis exacerbated by T3SS, we treated WT CR mice with bezafibrate, a Phosphatidylcholine synthesis inhibitor,^[^ ^24^ ^]^ and found bezafibrate significantly reduced serum phosphatidylcholine levels (Figure S4M, Supporting Information). Gross examination revealed that bezafibrate treatment significantly reduced atherosclerotic lesions in the aortic arch of mice receiving WT CR. Lipid deposition and plaque area in atherosclerotic lesions of WT CR‐treated mice were also significantly reduced by bezafibrate treatment, as evidenced by Oil red O and hematoxylin‐eosin staining of aortic roots (Figure 4F,G). In addition, bezafibrate treatment significantly reversed the decrease of smooth muscle cell content in plaques and the increase of macrophage infiltration in WT‐CR mice. Collectively, our data suggest that aberrantly upregulated T3SS virulence factors promote T2D‐AS progression, at least in part by causing abnormal increase in serum phosphatidylcholine, thereby exacerbating atherosclerosis. Importantly, butyrate administration can alleviate the deteriorating effect of WT‐CR on the development of atherosclerosis in T2D‐AS mice.

### 2.5 T3SS Damages the Intestinal Barrier by Inducing Ferroptosis of Intestinal Epithelial Cells in T2D‐AS Mice

**Figure 5:**
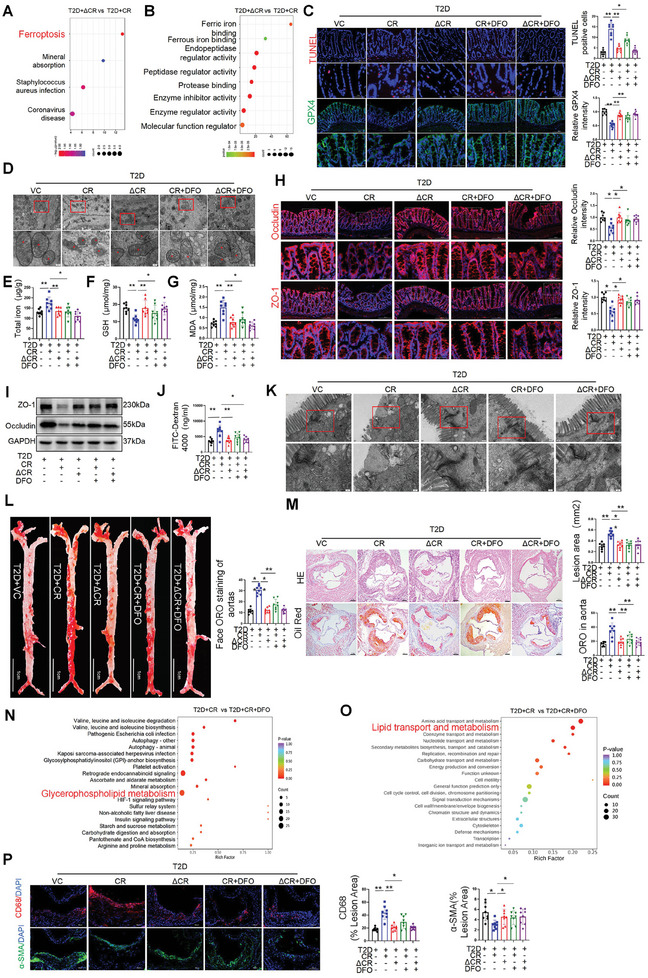
T3SS Damages the Intestinal Barrier by Inducing Ferroptosis of Intestinal Epithelial Cells in T2DM‐ASCVD Mice. A) Key protein and pathways regulated in Kyoto Encyclopedia of Genes and Genomes enrichment analysis. Mice were treated with gavage wild‐type CR (*n* = 3 mice) or ΔescN CR (*n* = 4 mice). B) Gene Ontology analysis of the significant proteins and their biological functions in mice treated with gavage wild‐type CR (*n* = 3 mice) or ΔescN CR (*n* = 4 mice). C) TUNEL staining of colonic epithelial cell ferroptosis. Red staining represents colonic epithelial cell ferroptosis. Representative immunofluorescence staining for GPX4 (*n* = 8 mice per group). Scale bars, 200 or 50 µm. D) Representative transmission electron microscope images of mitochondria in the group. Scale bars, 500 nm or 100 nm. E–G) The content of iron release, MDA, and GSH in intestinal tissue of mice (*n* = 8 mice per group). H) Representative immunofluorescence staining for ZO‐1 and occludin in mice fed with control, wild‐type CR, ΔescN CR, DFO treatment CR, and DFO treatment ΔescN CR. Immunofluorescence quantification of proteins in each group (*n* = 8 mice per group). Scale bars, 200 or 50 µm. I) Western blot analysis of the Occludin and ZO‐1 protein levels in the colon (*n* = 8 mice per group). J) Intestinal permeability assay: Plasma DX‐4000–FITC (mg/ml) oral challenge measured mice (*n* = 8 mice per group). K) Representative transmission electron microscope images of mitochondria in the group. Scale bars, 200 nm or 100 nm. L) Oil Red O staining of enface atherosclerotic lesions in aortas from indicated groups (*n* = 8 mice per group). Scale bars, 1 cm. M) Oil Red O staining on aortic root sections, and representative images of hematoxylin and eosin (HE) from indicated mice fed with control, wild‐type CR, ΔescN CR, DFO treatment CR, and DFO treatment ΔescN CR. Scale bars, 200 µm. N) Pathway enrichment analysis performed using the significantly different serum metabolites (*n* = 4 mice per group). O) Pathway enrichment analysis performed using the significantly different serum proteins (*n* = 4 mice per group). P) Immunostaining for smooth muscle actin (SMA) and F4/80 on aortic root slides collected from indicated mice. Quantification of atherosclerosis lesion areas in each group (*n* = 8 mice per group). Scale bars, 200 µm. All data are expressed as mean ± SEM, analyzed by an unpaired two‐sided t‐test. *P < 0.05, **P < 0.01, ***P < 0.001, ****P < 0.0001.

To understand how T3SS impairs the intestinal barrier, we conducted proteomics analysis of colon tissues from ΔescN CR‐ and WT CR‐infected mice. As shown in Figure S5A–C (Supporting Information), compared with that in ΔescN CR‐infected mice, eight proteins were upregulated and 48 proteins were downregulated in WT CR‐infected mice. KEGG analysis showed that differentially expressed proteins were enriched in multiple signaling pathways, including ferroptosis, mineral absorption, *Staphylococcus aureus* infection, and coronavirus disease (**Figure** **5**A). GO analysis revealed significant upregulation of ferric and ferrous iron‐binding signaling pathways in WT CR‐infected mice compared with that in ΔescN CR‐infected mice (Figure 5B). Next, we used TUNEL staining to explore the effect of T3SS on intestinal epithelial cell apoptosis,^[^ ^25^ ^]^ and found more TUNEL‐positive cells in the colonic tissues of the WT CR group than in those of the control group, which was reversed by administration of deferoxamine (DFO), a ferroptosis inhibitor.^[^ ^26^ ^]^ In contrast, compared with that in the control group, ΔescN CR, which lacks T3SS function, did not significantly increase the number of TUNEL‐positive cells and its effect was not affected by DFO treatment (Figure 5C). To further evaluate the occurrence of ferroptosis in the gut, we tested the samples for glutathione peroxidase 4 (GPX4), a key antioxidant enzyme and ferroptosis marker.^[^ ^27^ ^]^ Immunohistochemistry and immunofluorescence data showed that GPX4 expression was reduced in mice infected with WT CR compared with that in mice infected with vector control, whereas ΔescN CR did not affect GPX4 expression (Figure 5C; Figure S5D, Supporting Information).

We then performed TEM to detect the detailed structure of the mouse colon. As shown in Figure 5D, colon epithelial cells of mice infected with WT CR displayed mitochondrial pyknosis, dilated intercellular space. This differed from that observed in the control group, which showed intact colon epithelial barrier structure, normal mitochondrial morphology. In contrast, mitochondrial morphological characteristics of mice infected with ΔescN CR appeared to be similar to those of vector‐treated control mice. Furthermore, DFO supplementation significantly ameliorated WT‐CR‐infected‐induced damage in the mitochondrial morphological characteristics, intercellular intestinal barrier structure in mice (Figure 5D). The pathophysiological mechanism of ferroptosis involves iron accumulation.^[^ ^28^ ^]^ Total iron levels in the WT CR group were significantly higher than that in the control group. In addition, compared with those in the control group, glutathione (GSH) levels were significantly reduced, malondialdehyde levels were significantly increased in the WT CR group (Figure 5E–G). However, no difference in these parameters was detected between the ΔescN CR, the vector control group. Furthermore, administration of DFO to the WT CR‐infected mice increased GSH levels, reduced lipid peroxidation, and iron accumulation in the intestine (Figure 5E–G). Therefore, T3SS compromises the intestinal barrier, at least in part, by increasing ferroptosis in intestinal epithelial cells.

To further confirm these findings, we treated T2D‐AS mice with or without high T3SS abundance with DFO. Interestingly, DFO treatment attenuated the shortening of colon length (Figure S5D, Supporting Information) and reduced serum levels of FITC‐dextran in WT CR‐infected mice, but did not affect mice infected with ΔescN CR (Figure 4I). Consistently, DFO administration significantly increased the mRNA and protein levels of intestinal ZO‐1 and occludin in WT CR‐infected mice but not in ΔescN CR‐infected mice (Figure 5H–J; Figure S5E, Supporting Information). Furthermore, as shown in Figure 5K, supplementation with DFO substantially attenuated WT‐CR‐induced dilation of the intercellular space in the intestinal barrier. However, WT‐CR‐infected mice treated with DFO, like the control mice or mice infected with ΔescN CR, displayed a more complete and intact barrier structure. Furthermore, DFO administration significantly reduced atherosclerotic lesions, lipid deposition, and plaque area in WT CR‐infected mice but had no effect on ΔescN CR‐infected mice (Figure 5L,M; Figure S5F, Supporting Information).

We then performed serum metabolomics analysis to explore whether DFO could alleviate T3SS‐induced intestinal barrier damage and host metabolic changes. The PCA score map of serum metabolites showed that the metabolic composition of the WT CR group was significantly different from that of the WT CR plus DFO group (Figure S5G,H, Supporting Information). The main metabolites identified were glycerophospholipids, glycerides, and choline. KEGG pathway enrichment analysis showed that major pathways, including retrograde endocannabinoid signaling and glycerophospholipid metabolism, were enriched in WT CR plus DFO and WT CR groups. DFO treatment significantly reduced serum phosphatidyl ethanolamine, phosphatidylcholine, lysophosphatidyl ethanolamine, phosphatidylcholine, and lysophosphatidyl ethanolamine in WT CR mice (Figure 5N; Figure S5I, Supporting Information).

To further explore whether DFO alleviates intestinal barrier damage caused by T3SS, we performed proteomics on serum samples from the WT CR and WT CR plus DFO groups (Figure S5J,K, Supporting Information). KEGG enrichment analysis showed that differentially expressed proteins were enriched in various pathways, including lipid metabolism and transport and amino acid transport and metabolism. KEGG analysis revealed the effect of DFO treatment on the iron ion binding, 2 iron, 2 sulfur cluster binding metabolic pathways (Figure 5O). In addition, we found that proteins associated with tight junctions (such as Jip2 and Ext1) were significantly altered in the serum of mice treated with WT CR plus DFO compared with that in the WT CR‐infected mice. As shown in the heat map, DFO treatment significantly reduced the expression of serum pro‐inflammatory factors, such as MAPK and vascular cell adhesion protein 1, in the CR group (Figure S5L–O, Supporting Information). Macrophage content (CD68+ staining) in atherosclerotic lesions showed that DFO administration significantly reduced macrophage infiltration in mice infected with WT CR but not in mice infected with ΔescN CR (Figure 5P). These findings suggest that DFO can ameliorate T3SS‐induced dysregulation of glycerophospholipid metabolism and lipid metabolism in mice, while reducing systemic inflammation. In conclusion, our results showed that T3SS induces intestinal barrier damage by promoting ferroptosis and reducing the expression of occludin and ZO‐1 in intestinal epithelial cells, thereby aggravating atherosclerosis progression in T2D‐AS mice. This adverse effect can be ameliorated by inhibition of ferroptosis.

### 2.6 FTH1 Deficiency Reduces T3SS‐induced Ferroptosis of Intestinal Epithelial Cells and Inhibits the Damage to the Intestinal Barrier

**Figure 6:**
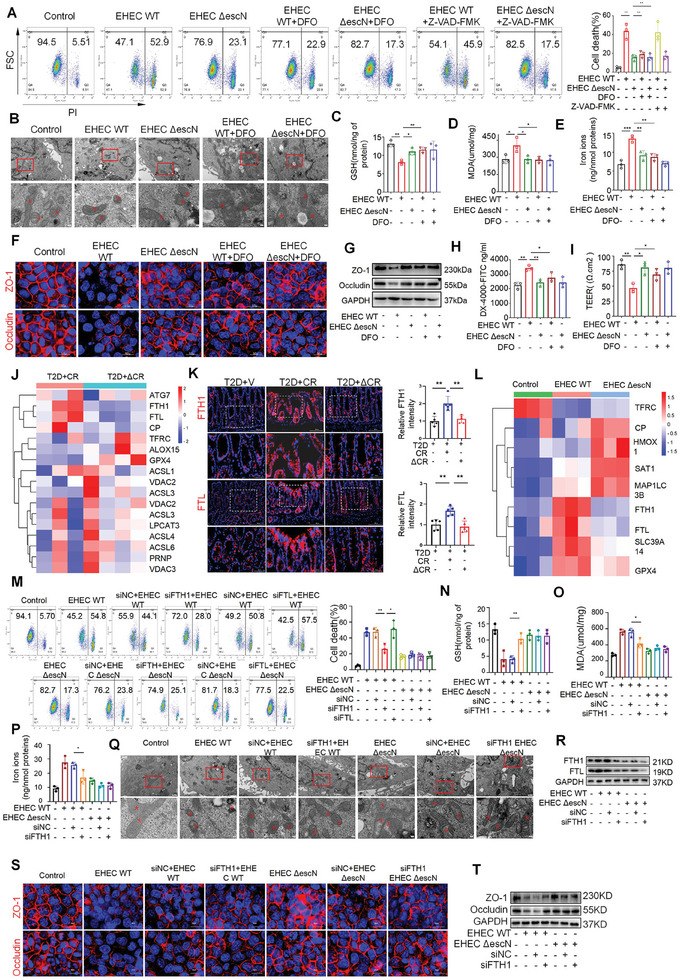
T3SS Damages the Intestinal Barrier by Inducing Ferroptosis in Human Colon Epithelial Cells. A) Analysis and quantification of cell viability by flow cytometry after different treatments (*n* = 3 per group). B) Representative transmission electron microscope images of mitochondria in the group (*n* = 3 per group). Scale bars, 500 or 100 nm. C–E) Iron release, MDA content, and GSH expression in cell (*n* = 3 per group). F) The expression of tight junction (TJ) proteins in HCoEpiC cell monolayers. Representative immunofluorescence image of ZO‐1 and occludin in intestinal epithelial cells (*n* = 3 per group).Scale bars, 50 µm. G) Western blotting images of ZO‐1 and occludin protein expression. Relative protein expression of ZO‐1 and occludin (*n* = 3 per group). H) FITC‐dextran fluorescence values in HCoEpiC cell monolayers (*n* = 3 per group). I) Transepithelial electrical resistance (TEER) (*n* = 3 per group). J) Hierarchical clustered heatmap of protein expression profiles for mice gavaged with CR (*n* = 3 mice) or with CR ΔescN (*n* = 4 mice). K) FTH1 and FTL immunofluorescence staining and quantification of colon (*n* = 8 mice per group). Scale bars, 200 or 50 µm. L) Heatmap mainly showing expression levels of up‐regulated and down‐regulated ferroptosis‐related genes in the HEC WT group, compared with those in the control group and EHEC ΔescN group (*n* = 3 per group). M) Analysis and quantification of cell viability using flow cytometry after different treatments (*n* = 3 per group). N–P) Iron release, MDA content, and GSH expression in cell lysates were detected using kits (*n* = 3 per group). Q) Representative TEM images of mitochondria in the Control, EHEC WT, siNC+EHEC WT, siFTH1+EHEC WT, EHEC ΔescN, siNC+EHEC ΔescN, and siFTH1+EHEC ΔescN groups (red arrowheads, classical mitochondria in the soma) (*n* = 3 per group). Scale bars, 500 or 100 nm. R) Western blot analyses of FTH1 and FTL proteins were performed (*n* = 3 per group). S) Representative immunofluorescence images of ZO‐1 and occludin in intestinal epithelial cells (*n* = 3 per group). Scale bars, 50 µm. T) The expression of tight junction (TJ) proteins in HCoEpiC cell monolayers. Western blotting images of ZO‐1 and occludin protein expression. Relative protein expression of ZO‐1 and occludin (*n* = 3 per group). All data are expressed as mean ± SEM, analyzed by an unpaired two‐sided t‐test. *P < 0.05, **P < 0.01, ***P < 0.001, ****P < 0.0001.

To further confirm these findings, we assessed the effect of T3SS on cultured human intestinal epithelial cells using a WT EHEC O157:H6 strain (WT EHEC) and a T3SS‐deficient strain (ΔescN EHEC).^[^ ^29^ ^]^ RNA sequencing(RNA‐seq) analysis of gene expression profiles revealed that uninfected, WT EHEC‐infected, and ΔescN EHEC‐infected cells exhibited distinct characteristics with 8478 differentially expressed genes (Figure S6A,B, Supporting Information). KEGG enrichment analysis showed that ferroptosis, autophagy, and apoptosis pathways were enriched with genes with altered expression in WT EHEC compared with those in the control or ΔescN EHEC (Figure S6C, Supporting Information). As shown in the heatmap analysis (Figure S6D,E, Supporting Information), the expression of ferritin‐ and apoptosis‐related genes was significantly altered in the WT EHEC group compared with those in the control group or the ΔescN EHEC group. This suggests that the presence and abundance of T3SS contribute significantly to the occurrence of ferroptosis and apoptosis. To verify this observation, we treated human colonic epithelial cells with an inhibitor of ferroptosis (DFO) or apoptosis (Z‐VAD‐FMK) in the presence and absence of WT EHEC or ΔescN EHEC. Inhibition of ferroptosis by DFO significantly ameliorated WT EHEC‐induced cell death, but inhibition of apoptosis by Z‐VAD‐FMK did not (**Figure** **6**A; Figure S6F,G, Supporting Information). These results indicate that WT EHEC‐induced cell death is caused by ferroptosis rather than by apoptosis.

Ferroptosis is characterized by changes in the ultramorphological structure of mitochondria.^[^ ^30^ ^]^ Indeed, we observed that WT EHEC caused significant mitochondrial morphological damages, including enhanced fragmentation and vanished cristae, compared to those in the control and ΔescN EHEC group (Figure 6B). These changes in mitochondrial morphology in the WT EHECs group were significantly improved by the ferroptosis inhibitor (Figure 6B). Ferroptosis typically depends on GSH depletion, increased lipid peroxidation, intracellular Fe^2+^ and intracellular iron accumulation, which were observed in cells treated with WT EHEC, compared with those in the control and ΔescN EHEC group (Figure 6C–E). Administration of DFO ameliorated these deleterious effects induced by WT EHEC. These findings indicate that T3SS promotes ferroptosis of intestinal epithelial cells.

Given the barrier function of the intestinal epithelium, we further investigated whether T3SS‐induced ferroptosis of intestinal epithelial cells would cause physical damage to the intestinal barrier. The tight junction proteins, ZO‐1 and occludin, which ensure intestinal barrier integrity, were tightly aligned with smooth edges in the control and ΔescN EHEC groups. However, after treatment with WT EHEC, the tight junction structure showed gaps. DFO administration increased the fluorescence intensity of ZO‐1 and occludin in WT EHEC‐treated cells, and tight junctions returned to a relatively intact and ordered structure (Figure 6F). Consistently, WT EHECs but not ΔescN EHECs, significantly reduced the levels of ZO‐1 and occludin, and the ferroptosis inhibitor mitigated this effect (Figure 6G). Consistently, as shown in Figure 6H,I, trans‐epithelial electrical resistance (TEER) was reduced, and the permeability for FITC‐dextran was increased in the WT EHEC group compared with that in the control group. Moreover, administration of the ferroptosis inhibitor, DFO, rescued the WT EHEC‐induced reduction in TEER and increase in permeability (Figure 6H,I). Together, these results implicate that T3SS in EHECs contributes to intestinal barrier dysfunction by promoting ferroptosis of intestinal epithelial cells.

Our proteomics data showed that ferroptosis‐associated proteins, ferritin heavy chain (FTH1)^[^ ^31^ ^]^ and ferritin light chain (FTL),^[^ ^32^ ^]^ were significantly upregulated in the intestine of WT CR‐infected mice compared with those in the ΔescN CR‐infected mice (Figure 6J). Compared with those in the control group, levels of FTH and FTL were consistently and significantly increased in the WT CR but not ΔescN CR group, as evidenced by western blots, immunofluorescence, and immunohistochemistry (Figure 6K; Figure S6H,K, Supporting Information). The mRNA levels of FTH1 and FTL were also significantly upregulated in the intestine of WT CR‐infected mice (Figure S6I,J, Supporting Information). RNA‐seq of intestinal epithelial cells also showed significant changes in the expression of ferroptosis‐related genes in pairwise comparisons of the control, WT EHEC, and ΔescN EHEC groups, among which FTH1 and FTL were significantly upregulated in the WT EHEC group compared with that in the vehicle control or ΔescN EHEC groups (Figure 6L). Consistently, qPCR analysis showed that only the mRNA level of FTH1 and FTL was significantly changed when comparing WT EHEC with control or ΔescN EHEC groups (Figure S6L, Supporting Information). The protein levels of FTH1 and FTL in the WT EHEC group were significantly higher than those in the control group, whereas ΔescN EHEC had no effect (Figure S6M, Supporting Information). To test the contribution of FTH1 and FTL to T3SS‐induced ferroptosis in intestinal epithelial cells, we used siRNA to knock down FTH1 and FTL expression in intestinal epithelial cells (Figure S6N–P, Supporting Information). As shown in Figure 6M, knockdown of FTH1 significantly reduced ferroptosis in WT EHEC‐infected epithelial cells but did not affect cell viability in ΔescN EHEC‐infected cells. However, FTL knockdown had no effect on cell death in either group, which was confirmed by propidium iodide(PI) staining (Figure S6Q, Supporting Information). Knockdown of FTH1 also reduced intracellular Fe^2+^, intracellular iron levels and lipid peroxidation, increased intracellular GSH levels, and corrected changes in mitochondrial morphology in cells infected with WT EHEC but not in cells infected with ΔescN EHEC (Figure 6N–Q). These data demonstrated that FTH1 deficiency can suppress T3SS‐induced ferroptosis in intestinal epithelial cells.

We then investigated the effect of FTH1 on T3SS‐induced intestinal barrier damage using FITC‐dextran flux. Knockdown of FTH1 significantly reduced FITC‐dextran permeability of cell monolayers in the WT EHEC group but did not affect permeability in the ΔescN EHEC group (Figure S6R, Supporting Information). Consistently, knockdown of FTH1 significantly increased TEER values in the WT EHEC group without affecting TEER values in the ΔescN EHEC groups, indicating that FTH1 deficiency rescues the WT EHEC‐induced damage in the intestinal barrier (Figure S6S, Supporting Information). Consistently, when FTH1 was knocked down, we observed increased expression of ZO‐1 and occludin in the WT EHEC group but not in the ΔescN EHEC group. Immunofluorescence of ZO‐1 and occludin also revealed that FTH1 deficiency restored their distribution and expression at cell‐cell junctions in the WT EHEC group (Figure 6R–T). In summary, these observations indicate that lack of FTH1 can attenuate T3SS‐induced intestinal epithelial cell ferroptosis and intestinal barrier damage.

### 2.7 FTH1 Knockdown Attenuates T3SS‐Induced Ferroptosis in Intestinal Epithelial Cells of T2D‐AS Mice, Restores the Intestinal Barrier, and Attenuates Atherosclerosis

**Figure 7:**
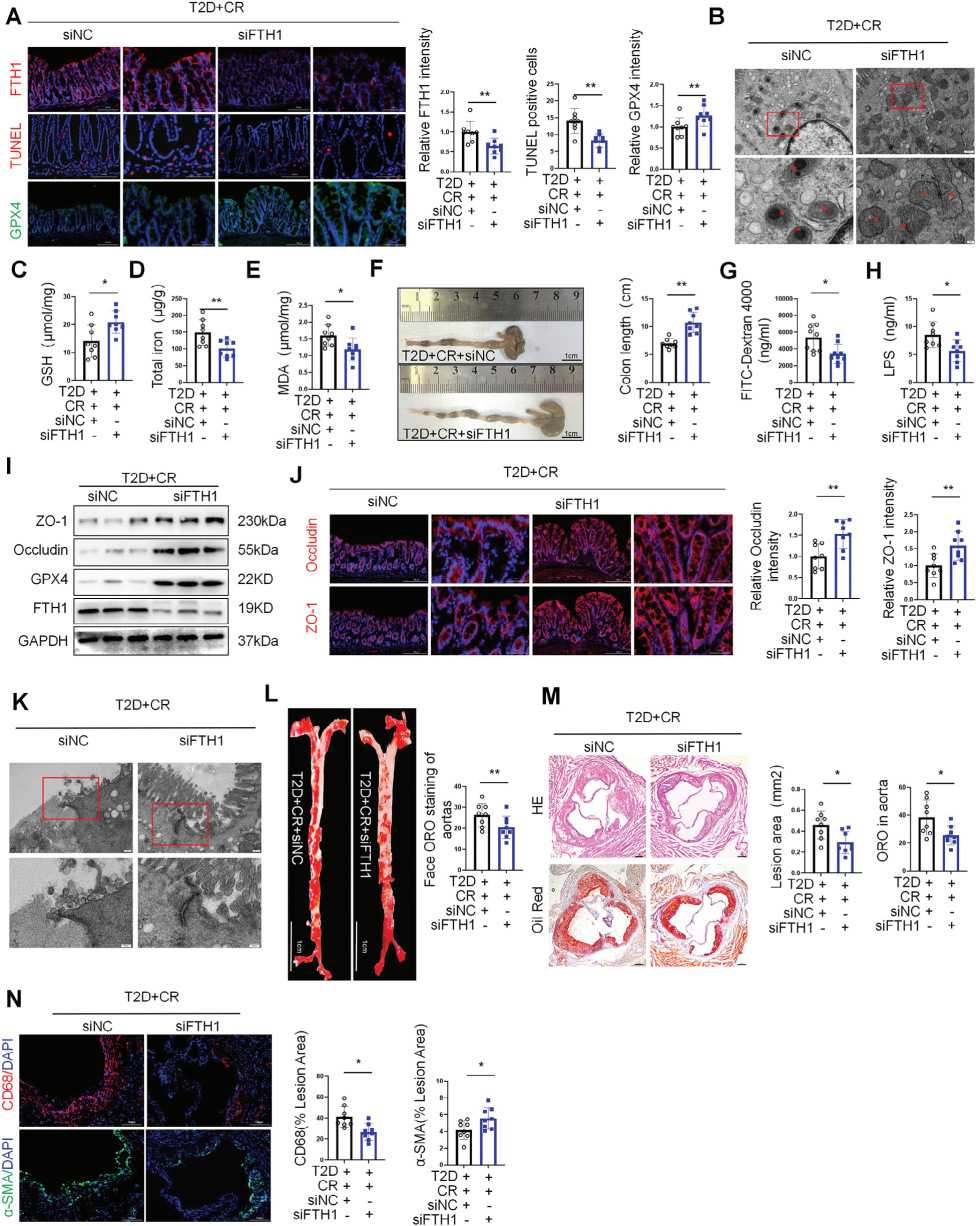
FTH1 knockdown attenuates T3SS‐induced ferroptosis in intestinal epithelial cells of T2D‐AS mice, restores the intestinal barrier, and attenuates atherosclerosis. A) TUNEL staining of colonic epithelial cell ferroptosis. Red staining represents colonic epithelial cell ferroptosis. Representative immunofluorescence staining for FTH1. Representative immunofluorescence staining for GPX4 (*n* = 8 mice per group). Scale bars, 200 or 50 µm. B) Representative transmission electron microscope images of mitochondria in the group (*n* = 8 per group). Scale bars, 500 or 100 nm. C–E) The content of iron release, MDA, and GSH in intestinal tissue of mice (*n* = 8 mice per group). F) Statistics of colon length (*n* = 8 mice per group). Scale bars, 1 cm. G) Intestinal permeability assay: Plasma DX‐4000–FITC (mg/ml) oral challenge measured mice (*n* = 8 mice per group). H) Serum LPS content of mice (*n* = 8 mice per group). I) Western blot analysis of the Occludin and ZO‐1 protein levels in the colon (*n* = 8 mice per group). J) Representative immunofluorescence staining for ZO‐1 and occludin staining of the colon tissue sections from the indicated groups of mice. (*n* = 8 mice per group). Scale bars, 200 or 50 µm. K) Representative transmission electron microscope images of mitochondria in the group (*n* = 8 per group). Scale bars, 200 or 100 nm. L) Oil Red O staining of enface atherosclerotic lesions in aortas from indicated groups (*n* = 8 mice per group). Scale bars, 1 cm. M) Oil Red O staining on aortic root sections, and representative images of hematoxylin and eosin (HE) from indicated mice fed with the indicated groups of mice. Scale bars, 200 µm. N) Immunostaining for smooth muscle actin (SMA) and F4/80 on aortic root slides collected from indicated mice. Quantification of atherosclerosis lesion areas in each group (*n* = 8 mice per group). Scale bars, 100 µm. All data are expressed as mean ± SEM, analyzed by an unpaired two‐sided t‐test. *P < 0.05, **P < 0.01, ***P < 0.001, ****P < 0.0001.

To evaluate whether FTH1 knockdown can reduce ferroptosis in T3SS‐induced intestinal epithelial cells of T2D‐AS mice, we knocked down the expression of FTH1 in colonic epithelial cells of 8‐week‐old mice by gavage with an adeno‐associated virus (AAV) vector encoding siFTH1. As shown, both mRNA and protein levels of FTH1 were down‐regulated in the colon tissues of AAV‐FTH1‐injected mice compared with the empty vector, and immunofluorescence staining confirmed the successful expression of AAV‐mediated GFP in the colon tissues (Figure S7A–C, Supporting Information). The siFTH1 group significantly reduced the number of tunnel‐positive cells in the colon tissues of WT CR‐infected mice compared with the empty vector group (**Figure** **7**A). to further assess the occurrence of ferroptosis in the intestine, we detected GPX4 in the colon tissues of WT CR‐infected mice, and WB and immunofluorescence data showed that, compared with the empty vector group, the siFTH1 group significantly restored the GPX4 expression (Figure 7A,I). In addition, a detailed structural examination of the mouse colon by transmission electron microscopy showed that the siFTH1 group significantly ameliorated the occurrence of mitochondrial sequestration and cellular gaps in the colonic epithelial cells of WT CR‐infected mice, as well as displaying a more intact and normalized mitochondrial morphology, compared with the empty vector group (Figure 7B). The siFTH1 group significantly reduced total iron and MDA levels in the colon tissues of WT CR‐infected mice compared with the empty vector group. In addition, the siFTH1 group significantly restored GSH levels in WT CR‐infected mice compared with the empty vector group. Thus, the knockdown of FTH1 could rescue T3SS‐induced ferroptosis in intestinal epithelial cells to a certain extent (Figure 7C–E).

To further confirm whether FTH1 knockdown could attenuate T3SS‐induced ferroptosis in the intestinal epithelial cells of T2D‐AS mice and rescue the damaged intestinal barrier. Interestingly, the results showed that siFTH1 attenuated colon length shortening and reduced intestinal permeability in WT CR‐infected mice, but the empty vector group did not affect WT CR‐infected mice (Figure 7F–H). Consistent with this, siFTH1 significantly increased the intestinal ZO‐1 and occludin protein levels in WT CR‐infected mice, but not in WT CR‐infected mice in the empty vector group (Figure 7I,J; Figure S7D, Supporting Information). Moreover, as shown in Figure 7K, the knockdown of FTH1 in colonic tissues greatly attenuated WT CR‐induced intestinal barrier cell gap expansion. Furthermore, macrophage content in atherosclerotic lesions (CD68+ staining) showed that siFTH1 significantly reduced macrophage infiltration in WT CR‐infected mice, but the empty vector group had no significant effect on WT CR‐infected mice (Figure 7L–N; Figure S7F, Supporting Information). But the results of changes in glycerophospholipid metabolism in intestinal epithelial cells with siRNA knocked down FTH1 and WT CR‐infected mice with siFTH1, and the results showed that there was no significant change in glycerophospholipid metabolism in intestinal epithelial cells of mice after FTH1 knockout (Figure S7G, Supporting Information). Taken together, our results suggest that knockdown of FTH1 expression in intestinal epithelial cells rescues T3SS‐induced ferroptosis in intestinal epithelial cells, restores the expression of occludin and ZO‐1 in intestinal epithelial cells, and ameliorates intestinal barrier injury, thereby attenuating the progression of atherosclerosis in T2D‐AS mice.

## 3 Discussion

Our findings reveal a previously unrecognized pathophysiological factor contributing to the increased severity of T2DM‐associated ASCVD. To date, research on the intersection of T2DM and dysregulation of gut microbiota has focused primarily on promoting atherogenesis through changes in metabolites (e.g., choline), oxidative stress, and inflammation.^[^ ^33^ ^]^ However, it is intuitive that intestinal microbiota undergoes both genomic and phenotypic changes during disease progression in the host organism. Our work highlights that the genomic virulence factors of a pathogen, not just its type, are major contributors to the heterogeneity in T2DM‐associated ASCVD.

This concept has the potential to inform the development of novel biomarkers, therapeutic targets. The inherent complexity of these biological systems renders the discovery of a single unifying mechanism highly improbable; it is unlikely that a single virulence factor can fully explain the severity of diabetes‐associated atherosclerosis. Mechanisms underlying a microbial shift toward enhanced virulence may be triggered by changes in host metabolism.^[^ ^34^ ^]^ We speculate that slow evolutionary selective pressures associated with repeated host‐pathogen interactions at the gut‐mucosal barrier inform these changes in a stochastic manner. Our analyses focusing on virulence factors, metagenomics identified numerous abundant T2DM‐ASCVD‐associated T3SS in the human gut microbiota. The mechanisms by which a microbial shift toward enhanced virulence aggravates ASCVD severity with T2DM progression have not yet been fully explored. Our results demonstrate that the *E. coli* genus is associated with T2DM‐ASCVD, which is consistent with observations in previous case–control studies of patients with symptomatic ASCVD. Obesity, diabetes, related manifestations are associated with enhanced hyperglycemia, which drives intestinal barrier dysfunction, increases the risk of enteric infection.^[^ ^35^ ^]^ Human intestinal *E. coli* pathogens mainly include EHEC, EPEC, which colonize the large, small intestines, respectively.^[^ ^36^ ^]^ Interestingly, we found a significant increase in the abundance of EHEC, EPEC in the gut microbiota of patients with T2DM‐ASCVD. More than 200 million cases of diarrhea caused by EPEC/EHEC infections are reported globally each year.^[^ ^37^ ^]^ These observations demonstrate that acute, noninvasive infections with attaching, effacing lesions may have profound impacts, particularly on the development of colon cancer, persistent colon infection, chronic colon inflammation.^[^ ^38^ ^]^ Various gut microbiota virulence factors belonging to iron uptake, T3SS, invasion classes are significantly associated with obesity.^[^ ^39^ ^]^ Here, we identified a high abundance of the virulence structure T3SS of *Enterobacteriaceae* in the gut microbiome of patients with T2DM, atherosclerosis. The potential role of intestinal T3SS virulence factors in diabetes with atherosclerosis is poorly understood. Emerging research suggests that intestinal barrier damage is a potential contributor to atherosclerosis.^[^ ^40^ ^]^ Here, we found that serum zonulin levels were elevated in patients with T2DM, atherosclerosis, were positively correlated with the severity of atherosclerosis. Oral administration of live *Citrobacter rodentium* to mimic the increase in intestinal T3SS virulence factors impaired the intestinal barrier, promoted the development of atherosclerosis in mice, highlighting the impact of pathogen virulence factors.

CR‐induced colitis in mice and EPEC/EHEC‐induced diarrheal symptoms in humans are generally considered transient disorders that are ameliorated by pathogen clearance.^[^ ^41^ ^]^ However, our results indicate that T3SS may have profound effects, particularly on the development of T3SS‐associated damage to the intestinal barrier. Uncontrolled ferroptosis in the intestinal epithelium is a potentially critical mediator in the development of intestinal barrier damage.^[^ ^27^ ^]^ Our findings reveal a novel pathway linking intestinal T3SS virulence factors, which orchestrate the FTH ferroptosis network, to intestinal barrier damage and exacerbation of atherosclerosis in diabetes. There are two main states of intracellular iron, one is in the form of Fe^2+^ which exists as an intracellular labile iron pool in the cell, Fe^2+^ is highly oxidizing and toxic and contributes to hydroxyl radical production.^[^ ^42^ ^]^ The second type is stored as Fe^3+^ bound to ferritin, including FTH1 and FTL, which impedes the accumulation of intracellular free iron by chelating with such ions.^[^ ^43^ ^]^ Infections such as those caused by the *Escherichia coli, Dengue virus* and *Mycobacterium tuberculosis* result in increased levels of ferritin,^[^ ^44^ ^]^ because of increased host cell uptake of iron.^[^ ^45^ ^]^ In addition, studies have shown that pathogen infection can directly affect the production of ferritin in host cells, where extracellular pathogen *E. coli* induces an iron retention phenotype in A549 cells,^[^ ^46^ ^]^ mainly characterized by the downregulation of the pivotal iron exporter ferroportin, the upregulation of the iron importer transferrin‐receptor‐1 and corresponding induction of the iron storage protein ferritin.^[^ ^46^ ^]^ In our research, intracellular Fe^2+^ levels were increased by T3SS, as evidenced by the substantial increase in in intracellular FerroOrange fluorescence intensity (Figure S6, Supporting Information). In our study, T3SS increased intracellular Fe^2+^ levels and upregulated intracellular FTH and FTL protein expression to induce ferroptosis in intestinal epithelial cells. This is contrary to previous studies, and we speculate that it may be that infection with the pathogen may potentially cause iron loading in intestinal epithelial cells, which in turn induces ferroptosis. The NRF2 signaling pathway also plays a key role in regulating ferroptosis‐related genes, including those involved in glutathione (GSH) synthesis (such as cysteine supply via SLC7A11, glutathione reductase, and GPX4), iron homeostasis (such as ferritin heavy chain (FTH) and ferritin light chain (FTL), transferrin receptor (TFRC), ferroportin (FPN), and heme oxygenase‐1 (HO‐1).^[^ ^47^ ^]^ Therefore, we hypothesized that T3SS may promote ferroptosis in intestinal epithelial cells by affecting NRF2, a key regulator of the ferroptosis process, thereby decreasing GPX4 expression and up‐regulating FTH1 expression to perturb cellular iron homeostasis. In future studies, we will use specific intestinal epithelial cell NRF2 knockout mice or specific inhibitors to investigate whether T3SS‐induced ferroptosis in intestinal epithelial cells plays a role by modulating NRF2, a key regulator in the process of ferroptosis. This suggests a new mechanism linking gut microbiota to the severity of T2DM‐related atherosclerosis, thereby opening potential avenues for novel therapies.

A large number of population studies have shown that serum phosphatidylcholine content is positively correlated with clinical atherosclerosis.^[^ ^48^ ^]^ For the first time, we show that an impaired intestinal barrier can lead to a disturbance of lipid metabolism, primarily through an increase in phosphatidylcholine. Recent studies have shown that glycerophospholipid metabolism plays a key role in the pathogenesis of AS and that disorders of glycerophospholipid metabolism directly affect the course of AS.^[^ ^49^ ^]^ Inhibition of phosphatidylcholine synthesis significantly attenuated atherosclerosis by ≈80% in Ldlr−/− mice.^[^ ^24d^ ^]^ The decrease of PC reduces the amounts of atherogenic lipoproteins by reducing VLDL secretion and increasing VLDL clearance from plasma. In our study, KEGG enrichment analysis revealed that glycerophospholipid metabolism may be an initiating factor in the T3SS‐induced development of T2DM‐ASCVD.These results help to further elucidate the pathogenesis of AS exacerbated by intestinal flora dysbiosis. In addition, chronic systemic inflammation caused by impaired intestinal barrier is not only related to bacterial translocation, but may be related to abnormally elevated phosphatidylcholine; however, long‐term research is required to demonstrate how this affects the progression of diabetes and the onset of atherosclerosis. In addition, our study focuses mainly on the *E. coli* T3SS. Notably, other bacterial species also have T3SS. Future studies using different causal inference methods are needed to determine whether the identified T3SS is causally related to atherosclerosis.

Recent studies have found that a high‐fiber diet interventions reduced the abundance of gut flora VF genes including T3SS.^[^ ^10^ ^]^ In addition, *Mucispirillum schaedleri* limited *Salmonella* infection, inhibited VF expression.^[^ ^50^ ^]^ Therefore, interference with intestinal VF expression should become novel methods to regulate the intestinal flora. Prior studies indicate short‐chain fatty acids, microbial metabolites at high concentrations in the gastrointestinal tract, limit population‐level T3SS‐1 gene expression.^[^ ^39^ ^]^ Butyric acid, as the main energy source of intestinal epithelial cells, plays an important role in the regulation, function of the intestinal barrier, its deficiency could lead to disruption of the gut barrier, cause multiple diseases.^[^ ^51^ ^]^ Here, our study confirms the role of butyrate in improving the intestinal barrier by inhibiting the virulent T3SS expression of enteric pathogens. This finding suggests that supplementation with butyrate may be of great significance in T2DM‐ASCVD treatment or regulation of intestinal flora. Numerous studies have found that salicylidene acylhydrazides, salicylanilides, sulfonyla minobenzanilides, benzimidazoles, thiazolidinone, some natural products to be effective against a number of pathogenic bacteria that utilize T3SS, including *Yersinia, Chlamydia, Salmonella, enteropatho genic E. coli, Shigella, Pseudomonas*.^[^ ^10^, ^52^ ^]^ These findings suggest that targeted inhibition of gut microbial T3SS function or manipulation of the microbiota through dietary interventions to inhibit intestinal T3SS virulence factors may improve gut health, mitigate the development of T2DM‐ASCVD. Furthermore, we have also discussed the fundamental importance of developing, maintaining healthy internal microbiome ecosystem, the intervention strategies for attaining beneficial microbial ecology, averting the pathogenic/T3SS overload. This provides new insights into the treatment of diabetes and atherosclerosis by modulating gut flora in clinical therapy.

In addition to its biological importance, our study may be relevant to the clinical management of patients with T2DM. Since the amount of intestinal T3SS virulence factors is associated with the risk of atherosclerosis, measurement of intestinal T3SS virulence factors may be a practical approach to predict patient outcomes. Furthermore, our data raise important clinical questions. Is the increased use of conventional antibiotics due to decreased resistance often found in patients with diabetes responsible for the increase in intestinal pathogenic bacteria and virulence factors?^[^ ^53^ ^]^ What causes the high incidence of chronic intestinal inflammatory and infectious disorders in some patients with diabetes? We suggest that patients with high levels of intestinal T3SS virulence factors should be treated with a combination of bacterial virulence inhibitors and intestinal barrier protective agents.

## 4 Experimental Section

### Human Samples

Ou of total 161 individuals enrolled, 103 were the patients with T2DM combined with ASCVD, while 58 served as age, gender, and BMI‐matched healthy controls (CON). Individuals with ASCVD showed clinical presentations of stable angina, unstable angina, or acute myocardial infarction (AMI) (Data S1, Supporting Information). ACVD diagnosis was confirmed by coronary angiography, and individuals that had ≥50% stenosis in single or multiple vessels were included. All patients were ethnic Han Chinese with no known consanguinity, aged 40–80 years old. Blood samples for clinical chemistry analyses were taken after an overnight fast for at least 10 h. Fasting or 2 h glucose, serum alanine aminotransferase (ALT), aspartate aminotransferase (AST), alkaline phosphatase (ALP) and γ‐glutamyl transpeptidase (GGT), TBIL, creatinine, uric acid, lipid profile, including triglycerides (TG), TC, high‐density lipoprotein cholesterol, and LDL cholesterol were measured using an autoanalyzer (Beckman Coulter AU5800). HBA1C was measured by high‐pressure liquid chromatography. T2DM patients were diagnosed based on the diagnostic criteria proposed by the World Health Organization (WHO) in 1999. Exclusion criteria were as follows: patients with special types of diabetes or gestational diabetes; tumor; liver, kidney, and other organs with severe damage; hyperhidrosis and other endocrine diseases; administration of antibiotics or probiotics in the past one month; diarrhea or other gastrointestinal diseases in the past one month; and history of gastrointestinal surgery. Informed consent was obtained from all participants. This study was approved by the Medical Ethics Committee of the Second Affiliated Hospital of Guilin Medical University (NO.YJS‐2021003). All procedures were performed in accordance with the principles of the Declaration of Helsinki.

### DNA Extraction and 16S rRNA Sequencing

Total genome DNA from samples was extracted using CTAB/SDS method. DNA concentration and purity was monitored on 1% agarose gels. According to the concentration, DNA was diluted to 1 ng µl^−1^ using sterile water. Primer:16S V4‐V5: 515F‐907R, 18S V9: 1380F‐1510R, ITS1: ITS1F‐ ITS2R. 16S /18S rRNA genes were amplified used the specific primer with the barcode. All PCR reactions were carried out in 30 µL reactions with 15 µL of PhusionHigh‐Fidelity PCR Master Mix (New England Biolabs); 0.2 µM of forward and reverse primers, and ≈10 ng template DNA. Thermal cycling consisted of initial denaturation at 98 °C for 1 min, followed by 30 cycles of denaturation at 98 °C for 10 s, annealing at 50 °C for 30 s, and elongation at 72 °C for 60 s. Finally, 72 °C for 5 min. Mix same volume of 1X loading buffer (contained SYB green) with PCR products and operate electrophoresis on 2% agarose gel for detection. Samples with bright main strip between 400–450 bp were chosen for further experiments. PCR products was mixed in Equi density ratios. Then, mixture PCR products was purified with GeneJET Gel Extraction Kit (Thermo Scientific). Sequencing libraries were generated using NEB NextUltraDNA Library Prep Kit for Illumina (NEB, USA) following manufacturer's recommendations and index codes were added. The library quality was assessed on the Qubit@ 2.0 Fluorometer (Thermo Scientific) and Agilent Bioanalyzer 2100 system. At last, the library was sequenced on an Illumina MiSeq platform and 250 bp/300 bp paired‐end reads were generated. Paired‐end reads from the original DNA fragments were merged using FLASH, a very fast and accurate analysis tool, which was designed to merge paired‐end reads when at least some of the reads overlap the read generated from the opposite end of the same DNA fragment. Paired‐end reads was assigned to each sample according to the unique barcodes.

### Animals

All apolipoprotein E–deficient mice were on C57BL/6 background. All mice were housed in a specific pathogen‐free animal facility at the Guilin Medical University Institute. The animals were fed normal chow diet or HFD and water ad libitum under a strict 12 h light/dark cycle. After 1 week of adaptive feeding, all mice were randomly assigned to the control (*n* = 8) and T2D group (*n* = 72). Following an intraperitoneal glucose tolerance test (IPGTT), mice in the T2D group were given an intraperitoneal injection of STZ (dissolved in 0.1 M citrate buffer, pH 4.5) once a day at a dose of 30 mg kg^−1^ body weight (BW) for three consecutive days. Meanwhile, mice in the control group received an intraperitoneal injection of 3 mL kg^−1^ BW citrate buffer (0.1 M, pH 4.5). After the three days of injections, the random blood glucose levels of all mice were detected once a day for another three consecutive days using a glucometer. The IPGTT was performed once again. Mice were gavaged with 100 µL AAV9‐FTH1 or AAV to enhance green fluorescent protein (GFP) at 1.00E + 12 v.g mL^−1^ (Shanghai Genechem Co., Ltd, China). Mice were randomly divided into treatment groups mice in the control group were gavaged with culture medium, those in the live *Citrobacter rodentium* (CR) group were gavaged with live CR, and those in the ΔescN CR group were gavaged with live ΔescN CR, those in the CR + butyrate treatment group were gavaged with live CR were administered with 200 mM butyrate in drinking water, those in the CR+DFO treatment group were gavaged with live CR were administered with 100 mM DFO in drinking water, those in the ΔescN CR + DFO treatment group were gavaged with live ΔescN CR were administered with 100 mM DFO in drinking water, CR and ΔescN CR at a dose of 2.5 × 109 cfu/100 µL of 1 times per week for 10 weeks. To validate CR clearance from infected mice, total DNA was extracted from mouse stools using the Quick‐DNA Fecal/Soil Microbe Kit (Zymo Research) and subjected to PCR using primers specific for CR espA and espF genes with sequences detailed in Table S3 (Supporting Information). Mice were terminated using i.p. injection of ketamine/xylazine (200 and 60 mg kg^−1^) followed by exsanguination via cardiac puncture. All experiments were performed according to the Guidelines for Animal Experiments in effect at Guilin Medical University (GLMC202105049) and followed EU guidelines (directive 2010/63/EU for the protection of laboratory animals).

### Bacterial Strains and Growth Conditions

Wild‐type C. rodentium (CR, DBS 100 strain), enterohemorrhagic *E. coli* O157:H7 (EHEC, EDL933 strain) and genetically manipulated bacterial strains used were summarized in Table S2 (Supporting Information). CR, EHEC strains were grown from single colonies on Luria–Bertani (LB) plates in LB broth at 37 °C overnight with shaking. For growth curve measurement, the overnight bacterial culture was diluted as indicated to grow in LB or DMEM medium at 37 °C with shaking, and the culture was taken at indicated time periods to measure absorbance at optical density 600 (OD600) on a POLARStar Omega Plate Reader (BMG Labtech, Cary, NC).

### Bacterial Infection In Vitro

Infection by EHEC and EHEC ΔescN in HCoEpiC cells was performed as previously described. Briefly, the indicated bacterial strain was washed with ice‐cold PBS and resuspended in prewarmed DMEM containing 10% heat‐inactivated FBS. Bacterial concentration was measured by absorbance at OD600, followed by a serial dilution and seeding on a MacConkey agar plate to confirm the administered CFU. HCoEpiC cells were infected by the indicated bacterial strains at a multiplicity of infection of 100 for indicated time periods, followed by whole‐cell lysis for immunoblotting or fixation for immunofluorescence staining, as previously described.

### Histology, IHC, and Immunofluorescence on Colon Tissue Sections

Histology, IHC, and immunofluorescence staining of colon tissue sections were performed as previously described. In brief, after euthanizing mice, the colons were removed under aseptic conditions and washed once with ice‐cold PBS, the colon was fixed in 10% buffered formalin for 24 h and frozen section, and 5‐µm sections were cut and processed for hematoxylin and eosin staining. Histopathology scores were determined in a blinded fashion using the following criteria as previously described. For immunostaining, the colon tissue sections were subjected to antigen retrieval in citrate buffer, pH 6.0 (Life Technologies). After blocking with appropriate sera and incubating with appropriate antibodies, sections were washed and incubated with either horseradish peroxidase–conjugated second antibodies, followed by DAB staining (Vector Laboratories), or fluorescence dye–conjugated second antibodies and 1 µg mL^−1^ DAPI (Sigma–Aldrich). All imaging was observed under a fluorescence microscope

### Immunofluorescence

For cellular immunofluorescence staining, cells were fixed with 4% paraformaldehyde in PBS and then mounted onto slides. After permeabilization with 0.05% Triton X‐100 in PBS and blocking with appropriate sera, the fixed cells were incubated with appropriate antibodies, counterstained with DAPI, and examined on an All imaging was observed under a fluorescence microscope.

### Quantitative Real‐Time PCR

Total RNA was isolated from colon tissues Or HCoEpiC cells using TRIzol reagent (Life Technologies) and treated with the TURBO DNA‐free Kit (Life Technologies) to remove residual genomic DNA. cDNA was synthesized using the ProtoScript First Strand cDNA Synthesis Kit (New England Biolabs) according to the manufacturer's instructions. Gene‐specific products were amplified using SsoAdvanced SYBR Green Supermix (Bio‐Rad Laboratories) using the primers with detailed sequences in Table S3 (Supporting Information)

### Mouse Intestinal Permeability Assay

Mice were administered 50 mg fluorescein isothiocyanate (FITC) dextran/100 g body weight via oral gavage 4 h prior to sacrifice. Immediately following euthanasia, blood was harvested via cardiac puncture, and the serum was subsequently separated via centrifugation. The serum from each animal was assayed for the presence and quantity of FITC signal with a TECAN Infinite M200 plate reader, using an excitation wavelength of 485 nm and an emission wavelength of 528 nm. A standard curve of FITC dextran was used to quantify the signal in each serum sample.

### Iron/GSH/MDA Assay

HCoEpiC cells were cultured and treated as described above. Cells are gently collected in PBS solution with a cell spatula. 1% Triton was used to permeabilize the cell membrane, and the cell lysates were centrifuged to obtain. The relative iron, GSH, and malondialdehyde (MDA) concentration in cell lysates were assessed via Iron Assay Kit (#A039‐2–1, Jiancheng, China), Glutathione Assay Kit (#A006‐2–1, Jiancheng, China) and MDA detection Kit (#A003‐4–1, Jiancheng, China) according to the manufacturer's instructions.

### Western Blot Analysis

The total proteins of colon tissues and cells were extracted by RIPA (#P0013C, Beyotime, China) with phosphatase inhibitor and PMSF (#P0012S, Beyotime, China). Immunoblotting was performed as previously described.6 Primary antibodies were as follows: GPX4 (1;1500), ZO‐1 (1;1500), Occludin (1:2000), FTH(1:2500), FTL (1:2000), GAPDH (1:2000). Secondary antibodies were as follows: peroxidase‐conjugated anti‐rabbit IgG (#2301, ZSGB‐BIO, China) and peroxidase‐conjugated anti‐mouse IgG (#2305, ZSGB‐BIO, China). The protein samples were visualized using the ECL‐chemiluminescent kit (#WBKLS0100, Millipore, USA) and analyzed by ImageJ software.

### Transmission Electron Microscopy (TEM)

Colon tissues and HCoEpiC cells was soaked in 2.5% glutaraldehyde and fixed in 1% osmium tetroxide for 1 h. It was then dyed in 2% uranyl acetate and dehydrated in graded ethanol. The samples Journal Pre‐proof were embedded in 100% acetone overnight at 4 °C, cut into 100 nm sections and stained with 2% lead citrate and uranyl acetate. The images were captured by transmission electron microscope.

### RNA Extraction, RNA Sequencing

mRNA with polyA tail was enriched by Oligo(dT) magnetic beads to obtain mRNA. mRNA was then broken into short fragments by fragmentation buffer. The short fragment RNA was used as template. The first strand CDNA was synthesized with six‐base random hexamers (random hexamers), then the two‐strand CDNA was synthesized by adding buffer, dNTPs (dUTP, dATP, dGTP, dCTP), DNA polymerase. AMPure XP beads were then used to purify double‐stranded CDNA. The purified double‐stranded CDNA was then end‐repaired, A tail was added, sequencing joints were connected. Then AMPure XP beads were used for fragment size selection, finally qPCR enrichment was performed to obtain the final CDNA library. After the completion of the library construction, the quality of the library was tested. 1) Qubit2.0 was used for preliminary quantification; Agilent 2100 was used to test the insert size of the library. The next experiment could be carried out only after the insert size met the expectation. The effective concentration of the library was accurately quantified by q‐PCR method (the effective concentration of the library was >2 mM) to complete the library inspection. Different libraries are pooled according to the target on‐machine data volume, Ilumina HiSeq platform was used for sequencing.

### NanoLCMS/MS Analysis

For each sample, 2 µg of total peptides were separated and analyzed with a nano­UPLC (EASY­nLC1200) coupled to a Q Exactive HFX Orbitrap instrument (Thermo Fisher Scientific) with a nano­electrospray ion source. Separation was performed using a reversed­phase column (100 µmID × 15 cm, Reprosil­Pur 120 C18­AQ, 1.9 µm, Dr. Maisch). Data dependent acquisition (DDA) was performed in profile and positive mode with Orbitrap analyzer at a resolution of 120 000 (at the rate of 200 m z^−1^) and m/z range of 350–1600 for MS1; For MS2, the resolution was set to 45 k with a fixed first mass of 110 m z^−1^. The top 20 most intense ions were fragmented by HCD with normalized collision energy (NCE) of 32%, and isolation window of 0.7 m z^−1^. Vendor's raw MS files were processed using Proteome Discoverer (PD) software (Version2.4.0.305) and the built‐in Sequent HT search engine. Peptide identification was performed with an initial precursor mass deviation of up to 10 ppm and a fragment mass deviation of 0.02 Da. Unique peptide and Razor peptide were used for protein quantification and total peptide amount for normalization. All the other parameters were reserved as default.

### Serum Metabolite Measurement, Analysis

16 serum samples from 16 individual mice were collected. Serum metabolites were extracted by mixing Chloroform:Methanol: Serum in 1:3:1 ratio, following centrifuged at 13 000 rpm for 3 min, supernatant was collected, performed LC‐ using an OrbitrapTM ExactiveTM mass spectrometer at the Glasgow Polyomics facility. Each metabolite was expressed by raw peak intensities, then, these peaks were analyzed step by step using R packages according to the method described in the previous studies (LCMS: An R package for automated semi targeted analysis in lipidomics). Generalized linear modeling, Pearson correlation were performed for normalized intensities of each metabolite across the different protein level, selected the significantly correlated metabolites (*p* < 0.05) into the IPA software to get the most affected pathways.

### Statistical Analysis

All statistical analyses were performed using GraphPad Prism 9.0 software. All data are presented as mean ± standard error of the mean. Statistical significance between two groups was determined by two‐tailed unpaired Student's t test when data were normally distributed, and nonparametric Mann–Whitney test was used when data were not normally distributed. One‐way ANOVA test was performed to compare the difference among multiple groups. Probability value of *p* < 0.05 was considered as statistically significant.

## Conflict of Interest

The authors declare no conflict of interest.

## Author Contributions

Y.Y.Z, S.T.C. and G.C. contributed equally to this work. Y.Y.Z, K.Y, S.T.C and X.Z developed the study concept and experimental design; G.C, L.Z, G.L.Z, X.Y.Y, L.Y, W.Q.D, Z.B.W, J.L, Y.F.T, D.W.Z, and Y.l., conducted the experiments; Y.Y.Z, A.S, X.Z, and K.Y wrote the manuscript, and the other co‐authors provided comments and revisions.

## Supporting information



Supporting Information

## Data Availability

The data that support the findings of this study are available in the supplementary material of this article.

## References

[1] H. Sun , P. Saeedi , S. Karuranga , M. Pinkepank , K. Ogurtsova , B. B. Duncan , C. Stein , A. Basit , J. C. N. Chan , J. C. Mbanya , M. E. Pavkov , A. Ramachandaran , S. H. Wild , S. James , W. H. Herman , P. Zhang , C. Bommer , S. Kuo , E. J. Boyko , D. J. Magliano , Diabetes Res. Clin. Pract. 2022, 183, 109119. 34879977 10.1016/j.diabres.2021.109119 PMC11057359

[2] a) J. A. Beckman , F. Paneni , F. Cosentino , M. A. Creager , Eur. Heart J. 2013, 34, 2444; 23625211 10.1093/eurheartj/eht142

[3] S. Sugimoto , H. A. Mena , B. E. Sansbury , S. Kobayashi , T. Tsuji , C. H. Wang , X. Yin , T. L. Huang , J. Kusuyama , S. D. Kodani , J. Darcy , G. Profeta , N. Pereira , R. E. Tanzi , C. Zhang , T. Serwold , E. Kokkotou , L. J. Goodyear , A. M. Cypess , L. O. Leiria , M. Spite , Y. H. Tseng , Nat. Metab. 2022, 4, 775. 35760872 10.1038/s42255-022-00590-0 PMC9792164

[4] A. Sharma , Y. Zheng , J. A. Ezekowitz , C. M. Westerhout , J. A. Udell , S. G. Goodman , P. W. Armstrong , J. B. Buse , J. B. Green , R. G. Josse , K. D. Kaufman , D. K. McGuire , G. Ambrosio , L. M. Chuang , R. D. Lopes , E. D. Peterson , R. R. Holman , Diabetes Care 2022, 45, 204. 34716214 10.2337/dc20-2806 PMC9004312

[5] a) M. Gurung , Z. Li , H. You , R. Rodrigues , D. B. Jump , A. Morgun , N. Shulzhenko , EBioMedicine 2020, 51, 102590; 31901868 10.1016/j.ebiom.2019.11.051 PMC6948163

[6] Z. Jie , H. Xia , S. L. Zhong , Q. Feng , S. Li , S. Liang , H. Zhong , Z. Liu , Y. Gao , H. Zhao , D. Zhang , Z. Su , Z. Fang , Z. Lan , J. Li , L. Xiao , J. Li , R. Li , X. Li , F. Li , H. Ren , Y. Huang , Y. Peng , G. Li , B. Wen , B. Dong , J. Y. Chen , Q. S. Geng , Z. W. Zhang , H. Yang , et al., Nat. Commun. 2017, 8, 845. 29018189 10.1038/s41467-017-00900-1 PMC5635030

[7] L. Zhang , Z. Wang , X. Zhang , L. Zhao , J. Chu , H. Li , W. Sun , C. Yang , H. Wang , W. Dai , S. Yan , X. Chen , D. Xu , Microbiol. Spectr. 2022, 10, e0032422. 35863004 10.1128/spectrum.00324-22 PMC9430528

[8] D. Ruano‐Gallego , J. Sanchez‐Garrido , Z. Kozik , E. Nunez‐Berrueco , M. Cepeda‐Molero , C. Mullineaux‐Sanders , Y. Naemi Baghshomali , S. L. Slater , N. Wagner , I. Glegola‐Madejska , T. I. Roumeliotis , T. Pupko , L. A. Fernandez , A. Rodriguez‐Paton , J. S. Choudhary , G. Frankel , Science 2021, 371. 10.1126/science.abc9531 33707240

[9] S. E. Ledwaba , D. V. S. Costa , D. T. Bolick , N. Giallourou , P. Medeiros , J. R. Swann , A. N. Traore , N. Potgieter , J. P. Nataro , R. L. Guerrant , Front Cell Infect. Microbiol. 2020, 10, 595266. 33392105 10.3389/fcimb.2020.595266 PMC7773950

[10] H. Li , G. Wu , L. Zhao , M. Zhang , Virulence 2021, 12, 1754. 34233588 10.1080/21505594.2021.1948252 PMC8274444

[11] C. Zhou , H. Zhao , X. Y. Xiao , B. D. Chen , R. J. Guo , Q. Wang , H. Chen , L. D. Zhao , C. C. Zhang , Y. H. Jiao , Y. M. Ju , H. X. Yang , Y. Y. Fei , L. Wang , M. Shen , H. Li , X. H. Wang , X. Lu , B. Yang , J. J. Liu , J. Li , L. Y. Peng , W. J. Zheng , C. Y. Zhang , J. X. Zhou , Q. J. Wu , Y. J. Yang , J. M. Su , Q. Shi , et al., J. Autoimmun. 2020, 107, 102360. 31806420 10.1016/j.jaut.2019.102360

[12] J. Zhao , H. Fan , T. Wang , B. Yu , S. Mao , X. Wang , W. Zhang , L. Wang , Y. Zhang , Z. Ren , B. Liang , Cardiovasc. Diabetol. 2022, 21, 123. 35778734 10.1186/s12933-022-01548-y PMC9250269

[13] a) E. Finlayson‐Trick , J. Nearing , J. A. Fischer , Y. Ma , S. Wang , H. Krouen , D. M. Goldfarb , C. D. Karakochuk , Microbiol. Spectr. 2023, 11, e0527322; 37199608 10.1128/spectrum.05273-22 PMC10269596

[14] A. Serapio‐Palacios , B. B. Finlay , Curr. Opin. Microbiol. 2020, 54, 67. 32058947 10.1016/j.mib.2019.12.001

[15] D. Truong , K. C. Boddy , V. Canadien , D. Brabant , G. D. Fairn , V. M. D'Costa , E. Coyaud , B. Raught , D. Perez‐Sala , W. S. Park , W. D. Heo , S. Grinstein , J. H. Brumell , Cell. Microbiol. 2018, 20, e12938. 30010242 10.1111/cmi.12938

[16] a) A. Cabal , S. Gomez‐Barrero , C. Porrero , C. Barcena , G. Lopez , R. Canton , C. Gortazar , L. Dominguez , J. Alvarez , Appl. Environ. Microbiol. 2013, 79, 4170; 23603685 10.1128/AEM.00537-13 PMC3697575

[17] N. M. Hanssen , O. Brouwers , M. J. Gijbels , K. Wouters , E. Wijnands , J. P. Cleutjens , J. G. De Mey , T. Miyata , E. A. Biessen , C. D. Stehouwer , C. G. Schalkwijk , Cardiovasc. Res. 2014, 104, 160. 25139743 10.1093/cvr/cvu189

[18] Y. Dai , Y. Shen , Q. R. Li , F. H. Ding , X. Q. Wang , H. J. Liu , X. X. Yan , L. J. Wang , K. Yang , H. B. Wang , Q. J. Chen , W. F. Shen , R. Y. Zhang , L. Lu , J. Am. Coll. Cardiol. 2017, 70, 2006. 29025558 10.1016/j.jacc.2017.08.053

[19] a) E. M. Mallick , M. E. McBee , V. K. Vanguri , A. R. Melton‐Celsa , K. Schlieper , B. J. Karalius , A. D. O'Brien , J. R. Butterton , J. M. Leong , D. B. Schauer , J. Clin. Invest. 2012, 122, 4012; 23041631 10.1172/JCI62746 PMC3484439

[20] B. R. Stevens , R. Goel , K. Seungbum , E. M. Richards , R. C. Holbert , C. J. Pepine , M. K. Raizada , Gut 2018, 67, 1555. 10.1136/gutjnl-2017-314759 PMC5851874 28814485

[21] C. Niu , X. L. Hu , Z. W. Yuan , Y. Xiao , P. Ji , Y. M. Wei , Y. L. Hua , J. Ethnopharmacol. 2023, 300, 115741. 36162543 10.1016/j.jep.2022.115741

[22] Z. Wang , E. Klipfell , B. J. Bennett , R. Koeth , B. S. Levison , B. Dugar , A. E. Feldstein , E. B. Britt , X. Fu , Y. M. Chung , Y. Wu , P. Schauer , J. D. Smith , H. Allayee , W. H. Tang , J. A. DiDonato , A. J. Lusis , S. L. Hazen , Nature 2011, 472, 57. 21475195 10.1038/nature09922 PMC3086762

[23] E. Nakamura , K. Maekawa , Y. Saito , T. Matsumoto , M. Ogawa , Y. Komohara , Y. Asada , A. Yamashita , PLoS One 2023, 18, e0281730. 36800352 10.1371/journal.pone.0281730 PMC9937458

[24] a) J. M. Jimenez‐Lopez , M. P. Carrasco , C. Marco , J. L. Segovia , Biochem. Pharmacol. 2006, 71, 1114; 16466701 10.1016/j.bcp.2005.08.001

[25] S. H. Ni , X. J. Zhang , X. L. OuYang , T. C. Ye , J. Li , Y. Li , S. N. Sun , X. W. Han , W. J. Long , L. J. Wang , Z. Q. Yang , L. Lu , Phytomedicine 2023, 115, 154807. 37121057 10.1016/j.phymed.2023.154807

[26] S. Yuan , C. Wei , G. Liu , L. Zhang , J. Li , L. Li , S. Cai , L. Fang , Cell Prolif. 2022, 55, e13158. 34811833 10.1111/cpr.13158 PMC8780895

[27] W. Gao , T. Zhang , H. Wu , Oxid. Med. Cell Longev. 2021, 2021, 4246255. 34733403 10.1155/2021/4246255 PMC8560274

[28] A. Anandhan , M. Dodson , A. Shakya , J. Chen , P. Liu , Y. Wei , H. Tan , Q. Wang , Z. Jiang , K. Yang , J. G. Garcia , S. K. Chambers , E. Chapman , A. Ooi , Y. Yang‐Hartwich , B. R. Stockwell , D. D. Zhang , Sci. Adv. 2023, 9, eade9585. 36724221 10.1126/sciadv.ade9585 PMC9891695

[29] L. Bzdzion , H. Krezel , K. Wrzeszcz , I. Grzegorek , K. Nowinska , G. Chodaczek , W. Swietnicki , Acta Biochim. Pol. 2017, 64, 49. 27864920 10.18388/abp.2016_1265

[30] H. Chen , Y. Qian , C. Jiang , L. Tang , J. Yu , L. Zhang , Y. Dai , G. Jiang , Biochim. Biophys. Acta Mol. Basis Dis. 2024, 1870, 166984. 38061600 10.1016/j.bbadis.2023.166984

[31] F. Yao , J. Peng , E. Zhang , D. Ji , Z. Gao , Y. Tang , X. Yao , X. Xia , Cell Death Differ. 2023, 30, 69. 35933500 10.1038/s41418-022-01046-4 PMC9883496

[32] J. Liu , Z. Ren , L. Yang , L. Zhu , Y. Li , C. Bie , H. Liu , Y. Ji , D. Chen , M. Zhu , W. Kuang , Cell Death Discov. 2022, 8, 99. 35249107 10.1038/s41420-022-00902-z PMC8898311

[33] G. Yang , J. Wei , P. Liu , Q. Zhang , Y. Tian , G. Hou , L. Meng , Y. Xin , X. Jiang , Metabolism 2021, 117, 154712. 33497712 10.1016/j.metabol.2021.154712

[34] J. F. Colbert , J. M. Kirsch , C. L. Erzen , C. J. Langouet‐Astrie , G. E. Thompson , S. A. McMurtry , J. M. Kofonow , C. E. Robertson , E. J. Kovacs , R. C. Sullivan , J. A. Hippensteel , N. V. Sawant , N. J. De Nisco , B. D. McCollister , R. S. Schwartz , A. R. Horswill , D. N. Frank , B. A. Duerkop , E. P. Schmidt , mBio 2023, 14, e0005223. 37102874 10.1128/mbio.00052-23 PMC10294665

[35] C. A. Thaiss , M. Levy , I. Grosheva , D. Zheng , E. Soffer , E. Blacher , S. Braverman , A. C. Tengeler , O. Barak , M. Elazar , R. Ben‐Zeev , D. Lehavi‐Regev , M. N. Katz , M. Pevsner‐Fischer , A. Gertler , Z. Halpern , A. Harmelin , S. Aamar , P. Serradas , A. Grosfeld , H. Shapiro , B. Geiger , E. Elinav , Science 2018, 359, 1376. 29519916 10.1126/science.aar3318

[36] J. L. Thomassin , J. R. Brannon , J. Kaiser , S. Gruenheid , H. Le Moual , Gut Microbes 2012, 3, 556. 22895086 10.4161/gmic.21656 PMC3495793

[37] C. Chuang , K. C. Lee , Y. P. Wang , P. C. Lee , T. E. Chang , Y. H. Huang , Y. T. Lin , M. C. Hou , Eur. J. Clin. Microbiol. Infect. Dis. 2023, 42, 1103. 37474764 10.1007/s10096-023-04644-3

[38] a) J. W. Collins , K. M. Keeney , V. F. Crepin , V. A. Rathinam , K. A. Fitzgerald , B. B. Finlay , G. Frankel , Nat. Rev. Microbiol. 2014, 12, 612; 25088150 10.1038/nrmicro3315

[39] A. M. Hockenberry , G. Micali , G. Takacs , J. Weng , W. D. Hardt , M. Ackermann , Proc. Natl. Acad. Sci. USA 2021, 118, e2103027118. 34330831 10.1073/pnas.2103027118 PMC8346864

[40] a) W. H. Tang , T. Kitai , S. L. Hazen , Circ. Res. 2017, 120, 1183; 28360349 10.1161/CIRCRESAHA.117.309715 PMC5390330

[41] E. L. Hartland , J. M. Leong , Front Cell Infect. Microbiol. 2013, 3, 15. 23641365 10.3389/fcimb.2013.00015 PMC3639409

[42] B. Hassannia , P. Vandenabeele , T. Vanden Berghe , Cancer Cell 2019, 35, 830. 31105042 10.1016/j.ccell.2019.04.002

[43] X. Fang , Z. Cai , H. Wang , D. Han , Q. Cheng , P. Zhang , F. Gao , Y. Yu , Z. Song , Q. Wu , P. An , S. Huang , J. Pan , H. Z. Chen , J. Chen , A. Linkermann , J. Min , F. Wang , Circ. Res. 2020, 127, 486. 32349646 10.1161/CIRCRESAHA.120.316509

[44] C. M. Gehrer , A. M. Mitterstiller , P. Grubwieser , E. G. Meyron‐Holtz , G. Weiss , M. Nairz , Int. J. Mol. Sci. 2023, 24, 4659. 36902088 10.3390/ijms24054659 PMC10003477

[45] K. A. Bauckman , I. U. Mysorekar , Autophagy 2016, 12, 850. 27002654 10.1080/15548627.2016.1160176 PMC4854542

[46] P. Grubwieser , A. Hoffmann , R. Hilbe , M. Seifert , T. Sonnweber , N. Bock , I. Theurl , G. Weiss , M. Nairz , Front Cell Infect. Microbiol. 2022, 12, 875543. 35663465 10.3389/fcimb.2022.875543 PMC9157649

[47] a) K. Shen , X. Wang , Y. Wang , Y. Jia , Y. Zhang , K. Wang , L. Luo , W. Cai , J. Li , S. Li , Y. Du , L. Zhang , H. Zhang , Y. Chen , C. Xu , J. Zhang , R. Wang , X. Yang , Y. Wang , D. Hu , Redox Biol. 2023, 62, 102655; 36913799 10.1016/j.redox.2023.102655 PMC10023991

[48] a) G. Lee , Y. S. Park , C. Cho , H. Lee , J. Park , D. J. Park , J. H. Lee , H. J. Lee , T. K. Ha , Y. J. Kim , S. W. Ryu , S. M. Han , M. W. Yoo , S. Park , S. U. Han , Y. Heo , B. H. Jung , Metabolomics 2021, 17, 71; 34355282 10.1007/s11306-021-01826-y

[49] V. T. Dang , L. H. Zhong , A. Huang , A. Deng , G. H. Werstuck , Metabolomics 2018, 14, 92. 30830446 10.1007/s11306-018-1392-2

[50] S. Herp , S. Brugiroux , D. Garzetti , D. Ring , L. M. Jochum , M. Beutler , C. Eberl , S. Hussain , S. Walter , R. G. Gerlach , H. J. Ruscheweyh , D. Huson , M. E. Sellin , E. Slack , B. Hanson , A. Loy , J. F. Baines , P. Rausch , M. Basic , A. Bleich , D. Berry , B. Stecher , Cell Host Microbe 2019, 25, 681. 31006637 10.1016/j.chom.2019.03.004

[51] A. L. Haber , M. Biton , N. Rogel , R. H. Herbst , K. Shekhar , C. Smillie , G. Burgin , T. M. Delorey , M. R. Howitt , Y. Katz , I. Tirosh , S. Beyaz , D. Dionne , M. Zhang , R. Raychowdhury , W. S. Garrett , O. Rozenblatt‐Rosen , H. N. Shi , O. Yilmaz , R. J. Xavier , A. Regev , Nature 2017, 551, 333. 29144463 10.1038/nature24489 PMC6022292

[52] H. A. Pendergrass , A. E. May , Antibiotics (Basel) 2019, 8, 162. 31554164 10.3390/antibiotics8040162 PMC6963908

[53] H. Q. Qu , Z. D. Jiang , Diabetes Res. Clin. Pract. 2014, 105, 285. 25015315 10.1016/j.diabres.2014.06.002

