# Supplementary material for: The Type III Secretion System (T3SS) of Escherichia Coli Promotes Atherosclerosis in Type 2 Diabetes Mellitus
Source: Adv Sci (Weinh). 2025 Jan 14;12(15):2413296. doi: 10.1002/advs.202413296 (PMC12005784; doi:10.1002/advs.202413296)
Supplement: Supplementary file 1 — Supporting Information [file ADVS-12-2413296-s001.docx]

**Graphical abstract** Proposed model of how the type III secretion system (T3SS) promotes atherosclerosis in type 2 diabetes mellitus. Higher *Enterobacteriaceae*/*Escherichia coli-Shigella* and T3SS abundance in T2DM patients with ASCVD. We use *Citrobacter rodentium* (CR) T3SS model to infect mice, induced in intestinal epithelial cell intracellular accumulation of iron ions by an abnormal increase in ferritin heavy chains , thereby resulting in ferroptosis and increasing intestinal permeability. Impairment of the intestinal barrier leads to disturbances in glycerophospholipid metabolism in mouse serum, including the high levels of phosphatidylcholine, activating macrophage as well as increased inflammation , and exacerbates atherosclerosis. Administration of butyrate or deferoxamine reversed the CR-induced increases in gut permeability, thereby preventing CR-T3SS-induced exacerbation of atherosclerotic lesion formation.

**Figure S1. The Abundance of T3SS of Pathogenic Escherichia Coli (E. Coli) Is Associated With Atherosclerosis Progression in Patients With T2DM**

(A) The taxonomic composition distribution among two groups on phylum-level of fecal microbiota (healthy controls (HC) (n=35) and patients with T2DM-ASCVD (n=45)).

(B) Principal Co-ordinates Analysis (PCoA) plots illustrating unweighted UniFrac distances among groups: HC (n=35) vs. T2DM-ASCVD (n=45).

(C) Venn diagram displaying that 1057 of 1309 OTUs were detected in the two groups, while 125 and 127 OTUs were unique to patients with T2DM-ASCVD (n=45) and HC (n=35), respectively.

(D) ANOVA histogram between genus level groups.

(E) Analysis using the R package, randforest.

(F) KEGG signaling pathways were compared between the placebo and psyllium husk groups (healthy controls (HC) (n=35) and patients with T2DM-ASCVD (n=45)).

(G) qRT-PCR analysis of the abundance of EHEC and EPEC in fecal samples(healthy controls (HC) (n=23) and patients with T2DM-ASCVD (n=58)).

**Figure S2. The T3SS of Pathogenic E. Coli Mediates the Aggravation of Atherosclerosis in T2DM**

(A) Relative abundance of gut microbiota at the genus level in the three groups. Relative abundance of gut microbiota at the species level in the three groups. Relative abundance of gut microbiota at the phylum level in the three groups. Relative abundance of gut microbiota at the family level in the three groups. Relative abundance of gut microbiota at the order level in the three groups. Relative abundance of gut microbiota at the class level in the three groups (n=6 mice per group).

(B) The relative abundance of gut microbiota at the genus level in the three groups was analyzed by bubble map. The relative abundance of gut microbiota at the species level in the three groups was analyzed by bubble map (n=6 mice per group).

(C) Relative abundance of *Akkermansiamuciniphila*, *Escherichia coli* (n=6 mice per group).

(D, E) Colony forming units (CFUs) recovered from stool and qRT-PCR analysis of the abundance of T3SS in mice fecal sample(n=8 mice per group).

(F) Serum lipid levels of mice (TC, TG, LDL-C, HDL-C) (n=8 mice per group). All data are expressed as mean ± SEM, analysed by an unpaired two-sided t-test. *P < 0.05, **P < 0.01, ***P < 0.001, ****P < 0.0001.

**Figure S3. Pathogenic E. Coli T3SS Causes Intestinal Barrier Damage to Accelerate Atherosclerosis in T2DM**

(A) Serum lipid levels of mice (TC, TG, LDL-C, HDL-C) (n=8 mice per group).

(B) qRT-PCR analysis to compare the expression of ZO-1 and occludin in colon tissue (n=8 mice per group).

(C) The expression of ZO-1 and occludin in colon tissue was analyzed using immunohistochemistry (n=8 mice per group). Scale bars, 200 μm. All data are expressed as mean ± SEM, analysed by an unpaired two-sided t-test. *P < 0.05, **P < 0.01, ***P < 0.001, ****P < 0.0001.

**Figure S4. T3SS Damages the Intestinal Barrier leads to abnormal phosphatidylcholine and lipid metabolism Accelerate Atherosclerosis in T2DM**

(A) PCA analysis of Non-target metabolomics serum samples (n=4 mice per group).

1. Volcano analysis of serum samples Non-target metabolomics data (n=4 mice per group).
2. Heatmap analysisof serum samples Non-target metabolomics data (n=4 mice per group).

(D) GO analysis of the metabolites in serum (n=4 mice per group).

(E) PCA analysis of Non-target proteomics serum samples (n=4 mice per group).

(F) Volcano analysis of TMT-MS proteomic data (n=4 mice per group).

(G) Heat map analysis of proteins related to lipid metabolism and transport (n=4 mice per group).

(H) Heat map analysis of proteins related to tight junction (n=4 mice per group).

(I) Heat map analysis of proteins related to inflammatory factor (n=4 mice per group).

(J) GO analysis of TMT-MS proteomic data (n=4 mice per group).

(K) Pathway enrichment analysis performed using the significantly different serum metabolites (n=4 mice per group).

(L) Changes in the relative expression of serum proteomic inflammatory factors(n=4 mice per group).

(M) Lipids were extracted from plasma (100 μL) , and the amount of PC were measured by phosphorus assay (n=8 mice per group). All data are expressed as mean ± SEM, analysed by an unpaired two-sided t-test. *P < 0.05, **P < 0.01, ***P < 0.001, ****P < 0.0001.

**Figure S5. T3SS Damages the Intestinal Barrier by Inducing Ferroptosis of Intestinal Epithelial Cells in T2DM-ASCVD Mice**

1. Principal component analysis (PCA) of TMT-MS proteomic data.
2. Volcano plot displays differentially expressed proteins in gavage wild-type CR mice (n=3 mice) compared with those in gavage ΔescN CR mice (n=4 mice).

(C) Hierarchical clustered heatmap of protein expression profiles for mice treated with gavage wild-type CR (n=3 mice) or ΔescN CR (n=4 mice).

(D) Statistics of colon length (n=8 mice per group). Scale bars, 1 cm.

(E) The expression of GPX4 in colon tissue was analyzed using immunohistochemistry (n=8 mice per group). Scale bars, 200 μm.

(F) The expression of ZO-1 and occludin in colon tissue was analyzed using immunohistochemistry (n=8 mice per group).

(G) PCA analysis of Non-target metabolomics serum samples (n=4 mice per group).

(H) Volcano analysis of serum samples Non-target metabolomics data (n=4 mice per group).

(I) Non-target metabolomics serum samples heatmap (n=4 mice per group).

(J) PCA analysis of Non-target proteomics serum samples (n=4 mice per group).

(K) Volcano analysis of TMT-MS proteomic data (n=4 mice per group).

(L) Heatmap analysisof TMT-MS proteomic data (n=4 mice per group).

(M) Heat map analysis of proteins related to lipid metabolism and transport (n=4 mice per group).

(N) Heat map analysis of proteins related to tight junction (n=4 mice per group).

(O) Heat map analysis of proteins related to inflammatory factor (n=4 mice per group).

(P) Serum LPS content of mice (n=8 mice per group).

(Q) Serum lipid levels of mice (TC, TG, LDL-C, HDL-C) (n=8 mice per group).

(R) Scatterplots indicating correlation of iron release, MDA, and GSH in intestinal tissue with enface atherosclerotic lesions in aortas (n=40). Correlation coefficients are reported as Spearman correlations. All data are expressed as mean ± SEM, analysed by an unpaired two-sided t-test. *P < 0.05, **P < 0.01, ***P < 0.001, ****P < 0.0001.

**Figure S6. T3SS Damages the Intestinal Barrier by Inducing Ferroptosis in Human Colon Epithelial Cells**

(A) Principal component analysis (PCA) of transcriptomics data (n=3 per group).

(B) Venn diagram representation of up-regulated genes (n=3 per group).

(C) Kyoto Encyclopedia of Genes and Genomes (KEGG) enrichment analysis was performed to identify the most significantly altered signaling pathways in the present EHEC WT group, compared with those in the control group and EHEC ΔescN group (n=3 per group).

(D) Heatmap mainly showing expression levels of up-regulated and down-regulated apoptosis-related genes in the HEC WT group, compared with those in the control group and EHEC ΔescN group (n=3 per group).

(E) Heatmap mainly showing expression levels of up-regulated and down-regulated autophagy-related genes in the HEC WT group, compared with those in the control group and EHEC ΔescN group (n=3 per group).

(F) Cell nuclei were visualized by staining with DAPI, actin by staining with Bodipy-phalloidin, and bound bacteria by anti-EHEC O157-FITC antibody (n=3 per group). Scale bars, 100 μm.

(G) Representative images of intestinal HCoEpiC cells stained by PI immunofluorescence (n=3 per group). Scale bars, 1000 μm. Detection of intracellular Fe2+ content in cells using FerroOrange fluorescent probe (n=3 per group). Scale bar = 50 μm.

(H) Representative images of immunohistochemistry staining for FTH1 and FTL (n=8 mice per group). Scale bars, 200 μm.

(I, J) The relative mRNA levels ofFTH1 and FTL in colon tissue (n=8 mice per group).

(K) Western blot analysis ofFTH and FTL proteins in colon tissues (n=8 mice per group).

(L) The relative mRNA levels of FTH, FTL, SLC39a14, GPX4, CP, HMOX1, and SAT1 in HCoEpiC cells (n=3 per group).

(M) Western blot analyses of FTH and FTL proteins were performed (n=3 per group).

(N) HCoEpiC cells were transfected with negative control siRNA (si-NC) or specific FTH siRNA (si-FTH) for 48 h. The mRNA levels ofFTH after FTH siRNA transfection (n=3 per group).

(O) The protein levels ofFTH in HCoEpiC cells after FTH siRNA transfection.

(P) HCoEpiC cells were transfected with negative control siRNA (si-NC) or specific FTH siRNA (si-FTL) for 48 h. The mRNA levels ofFTL after FTL siRNA transfection (n=3 per group). The protein levels of FTL in HCoEpiC cells after FTL siRNA transfection (n=3 per group).

(Q). Representative images of intestinal HCoEpiC cells stained using PI immunofluorescence (n=3 per group). Scale bars, 1000 μm.

(R) FITC-dextran fluorescence values in HCoEpiC cell monolayers (n=3 per group).

(S) Transepithelial electrical resistance (TEER) (n=3 per group). Scale bars, 500 nm.

(T) Detection of intracellular Fe2+ content in cells using FerroOrange fluorescent probe (n=3 per group). Scale bar = 50 μm. All data are expressed as mean ± SEM, analysed by an unpaired two-sided t-test. *P < 0.05, **P < 0.01, ***P < 0.001, ****P < 0.0001.

**Figure S7. FTH1 knockdown attenuates T3SS-induced ferroptosis in intestinal epithelial cells of T2D-AS mice, restores the intestinal barrier, and attenuates atherosclerosis**

(A) Western blot analysis of FTH1 proteins in colon tissues (n=8 mice per group).

(B) The mRNA levels of FTH1 after AAV-FTH1 transfection (n=8 mice per group).

(C) Representative immunofluorescence staining for FTH1 and GFP staining of the colon tissue sections from the indicated groups of mice. (n=8 mice per group). Scale bars, 200 μm.

(D) The expression of FTH1, GPX4, ZO-1 and occludin in colon tissue was analyzed using immunohistochemistry (n=8 mice per group). Scale bars, 200 μm.

(E) Serum lipid levels of mice (TC, TG, LDL-C, HDL-C) (n=8 mice per group).

(F) Scatterplots indicating correlation of iron release, MDA, and GSH in intestinal tissue with enface atherosclerotic lesions in aortas (n=8). Correlation coefficients are reported as Spearman correlations.

(G) Colon tissues of mice were collected and, and the amount of PC were measured by phosphorus assay (n=8 mice per group). All data are expressed as mean ± SEM, analysed by an unpaired two-sided t-test. *P < 0.05, **P < 0.01, ***P < 0.001, ****P < 0.0001.

**Graphical abstract** Proposed model of how the type III secretion system (T3SS) promotes atherosclerosis in type 2 diabetes mellitus. Higher *Enterobacteriaceae*/*Escherichia coli-Shigella* and T3SS abundance in T2DM patients with ASCVD. We use *Citrobacter rodentium* (CR) T3SS model to infect mice, induced in intestinal epithelial cell intracellular accumulation of iron ions by an abnormal increase in ferritin heavy chains , thereby resulting in ferroptosis and increasing intestinal permeability. Impairment of the intestinal barrier leads to disturbances in glycerophospholipid metabolism in mouse serum, including the high levels of phosphatidylcholine, activating macrophage as well as increased inflammation , and exacerbates atherosclerosis. Administration of butyrate or deferoxamine reversed the CR-induced increases in gut permeability, thereby preventing CR-T3SS-induced exacerbation of atherosclerotic lesion formation.

**Table S1 KEGG.pathway**

| **Pathway Id** | **Pathway Level1** | **Pathway Level2** | **Pathway Level3** | **HC(n=35)** | **T2DM-ASCVD(n=45)** | **p-value** |
| --- | --- | --- | --- | --- | --- | --- |
| **ko05100** | **Human Diseases** | **Infectious disease: bacterial** | **Bacterial invasion of epithelial cells** | **424.61±472.31** | **5047.41±6049.90** | **P < 0.05** |
| ko05111 | Cellular Processes | Cellular community - prokaryotes | Biofilm formation - Vibrio cholerae | 14293.87±4810.30 | 15747.15±3444.06 | P > 0.05 |
| ko05120 | Human Diseases | Infectious disease: bacterial | Epithelial cell signaling in Helicobacter pylori infection | 19425.14±4701.14 | 18661.10±3211.95 | P > 0.05 |
| **ko05130** | **Human Diseases** | **Infectious disease: bacterial** | **Pathogenic Escherichia coli infection** | **22.71±33.90** | **335.37±417.70** | **P < 0.05** |
| ko05131 | Human Diseases | Infectious disease: bacterial | Shigellosis | 295.82±440.40 | 4382.25±5485.26 | P < 0.05 |
| ko05142 | Human Diseases | Infectious disease: parasitic | Chagas disease (American trypanosomiasis) | 169.92±206.78 | 847.22±715.11 | P > 0.05 |
| ko05143 | Human Diseases | Infectious disease: parasitic | African trypanosomiasis | 198.32±211.43 | 892.81±729.72 | P < 0.05 |
| ko05145 | Human Diseases | Infectious disease: parasitic | Toxoplasmosis | 52.46±54.71 | 65.18±205.64 | P > 0.05 |
| ko05146 | Human Diseases | Infectious disease: parasitic | Amoebiasis | 1045.16±897.92 | 1408.37±882.97 | P > 0.05 |
| ko05150 | Human Diseases | Infectious disease: bacterial | Staphylococcus aureus infection | 2538.48±5614.14 | 3665.53±5875.39 | P > 0.05 |

Data are presented as mean ± SD affected.

**Table S2 VFDB virulence Factor Notes**

| **VFS** | **Species** | **HC (n=6)** | **T2D-AS (n=6)** | **AS (n=6)** |
| --- | --- | --- | --- | --- |
| LEE encoded T3SS | E.coli | 0.00 | 3.28**±**5.92 | 0.00 |
| LEE encoded T3SS | E.coli | 0.00 | 0.59**±**1.26 | 0.01 |
| LEE encoded T3SS | E.coli | 0.00 | 4.89**±**7.65 | 0.00 |
| LEE encoded T3SS | E.coli | 0.00 | 2.67**±**3.55 | 0.00 |
| LEE encoded T3SS | E.coli | 0.00 | 1.97**±**2.93 | 0.03**±**0.07 |
| T3SS | C.rodentium | 0.00 | 4.57**±**7.23 | 0.00 |
| T3SS | C.rodentium | 1.00**±0.59** | 2.71**±**5.14 | 2.21**±**2.95 |
| T3SS | C.rodentium | 0.00 | 6.69**±**7.83 | 0.24**±**0.40 |

Data are presented as mean ± SD affected.
